# Quality assessment of clinical practice guidelines using the AGREE instrument in Japan: A time trend analysis

**DOI:** 10.1371/journal.pone.0216346

**Published:** 2019-05-02

**Authors:** Kanako Seto, Kunichika Matsumoto, Shigeru Fujita, Takefumi Kitazawa, Rebeka Amin, Yosuke Hatakeyama, Tomonori Hasegawa

**Affiliations:** 1 Department of Social Medicine, Toho University School of Medicine, Tokyo, Japan; 2 Faculty of Health Sciences, Tokyo Kasei University, Saitama, Japan; 3 Department of Social Medicine, Toho University Graduate School of Medicine, Tokyo, Japan; Universitat Witten/Herdecke, GERMANY

## Abstract

**Background:**

Clinical practice guidelines (CPGs) are representative methods for promoting the standardization of healthcare and improvement of its quality. Few studies have investigated changes in the quality of CPGs published in a country over time. Our aim was to investigate changes in the quality of CPGs over time in the context of the available infrastructure for CPG development, public interest in healthcare quality, and healthcare providers’ responses to this interest.

**Methods:**

All CPGs pertaining to evidence-based medicine (EBM) issued between 2000 and 2014 in Japan (n = 373) were evaluated using the Japanese version of the Appraisal of Guidelines for Research and Evaluation (AGREE) I. Additionally, time trends in quality were analyzed. Using a cut-off point based on the publication year of CPG development literature, the evaluated CPGs were classified into those published until 2008 (pre-2008) and those published since 2009 (post-2008). Subsequently, we compared these groups in terms of 1) first edition CPGs and its second editions, and 2) patients’ version of CPGs.

**Results:**

Scores on all six domains of AGREE I improved each year. A comparison of the first- and second-edition of CPGs (n = 64) showed that scores on all domains improved significantly after revision. Significant improvement was observed in three domains (#2 stakeholder involvement, #3 rigor of development, and #4 clarity of presentation) in the pre-2008 group and in all domains in the post-2008 group. The comparison between the pre- and post-2008 groups in terms of CPGs for patients showed that the score increased in only one domain (#1 scope and purpose).

**Conclusions:**

The number of published CPGs has been increasing and the quality of CPGs, as assessed using the AGREE I instrument, has been improving. These changes seem to be influenced by improvements in social infrastructure, such as the publication of CPG development procedures, availability of CPG preparation methodology training, and increase in CPG-related skills.

## Introduction

Clinical practice guidelines (CPGs) are representative methods for promoting the standardization of healthcare and improvement of its quality. Since 2000, the Japan Ministry of Health, Labour and Welfare (MHLW) has encouraged academic societies in Japan to develop CPGs for major diseases using public research funds. Currently, academic societies and research groups are involved in developing and managing CPGs, and approximately 30–40 CPGs, including newly developed and revised CPGs, are being issued each year. Additionally, infrastructure has been developed to facilitate CPG publication. The Japan Council for Quality Health Care (JQ) released a handbook on CPG development to standardize preparation methods and thus facilitate the development of CPGs [1,2]. The Toho University Medical Media Center, Japan Medical Abstracts Society [3], and JQ Medical Information Network Distribution Service also maintain a clearinghouse of CPGs [4].

The Appraisal of Guidelines for Research and Evaluation (AGREE) is a quality-assessment tool focusing on CPG preparation methodology [5,6]. It was developed by the AGREE collaboration. Two editions of the tool, AGREE I and II [7,8], have been translated to over 20 languages [9]. By clarifying CPG evaluation criteria, AGREE intended to promote the efficient preparation of high-quality CPGs.

Several studies have evaluated CPGs for specific diseases using AGREE [10–14]. However, these studies only focused on specific diseases or specific periods, and only a few studies have investigated the impact of changes in CPGs published in one country over time or compared CPGs before and after their revision. Changes in the quality of CPGs over time reflect healthcare standardization, public interest in healthcare quality, and healthcare providers’ responses to this interest.

The present study aimed to use the Japanese version of AGREE I to evaluate CPGs on evidence-based medicine (EBM) developed in Japan. We also compared CPGs before and after revision, and compared CPGs for patients published until 2008 and those published since 2009. Our aim was to investigate changes in the quality of CPGs over time in the context of the available infrastructure for CPG development, public interest in the quality of healthcare, and healthcare providers’ responses to this interest.

## Methods

In the Toho University Medical Media Center, which has been managing the Japanese guidelines clearinghouse since 2001, medical librarians searched and collected potentially relevant Japanese literature from all literatures published in Japan. Subsequently, experienced medical librarians screened and selected CPGs for the quality assessment based on the following predefined criteria: (1) the title includes the term “guideline,” “shishin” (guidance), or “tebiki” (guide), (2) the methodology describes the CPG development process based on EBM, and (3) the theme relates to clinical practice, and not to topics such as medical ethics or animal experimentation. Three hundred and seventy-three EBM-based CPGs were identified between 2000 and 2014 (S1 Table).

They were independently evaluated by three librarians using the Japanese version of AGREE I [15]. We have been developing a database on CPG evaluation using AGREE I since 2001. AGREE II, the updated version of AGREE I [7], was published in 2009 [8]. CPG evaluation has been conducted using AGREE II along with AGREE I, and the results of the former have been registered in the database since 2011. Additionally, the implementation of evaluation tools for CPGs may affect the quality improvement of CPGs. Therefore, to analyze the long-term trend in the quality of CPGs, including the influence of implementation of AGREE II, we used results of the evaluation conducted using AGREE I (S2 Table).

AGREE I comprises 23 specific items and one overall assessment item. The items are categorized into the following six domains: #1: scope and purpose, #2: stakeholder involvement, #3: rigor of development, #4: clarity of presentation, #5: applicability, and #6: editorial independence. CPGs were evaluated independently by three evaluators, using a 4-point Likert scale (1 = strongly disagree and 4 = strongly agree). A standardized score was calculated for each domain according to the AGREE manual [15]. The three CPG evaluators were members of the evaluation group, which comprised four librarians with experience in CPG development and evaluation.

A standardized score for each domain was calculated according to the following formula:

[(Obtainedscore−Minimumpossiblescore)(Maximumpossiblescore−Minimumpossiblescore)]×100[%]

For the time trend analysis, standardized scores for the six domains were calculated for every 2 years (the score for 2000 included EBM-based CPGs issued until 2000), and the Kruskal-Wallis test was used to examine differences.

Around 2009, a circumstances of development of Japanese CPGs changed drastically. AGREE II was issued in 2009 [8]. The JQ published the handbook for Japanese CPG development in 2007 [1], “which served as the basis for the development of CPGs in Japan for considerable time” [16]. It is reasonable to assume a delay of a few years from the publication of the handbook to the influence on completed CPGs. Therefore, for subsequent analyses, we divided selected CPGs into those published until 2008 (pre-2008) and those published since 2009 (post-2008).

To analyze the quality of CPGs before and after the revision, pairs of first and second edition CPGs were extracted (n = 64) and their median scores were examined using the Wilcoxon signed-rank test. Second-edition CPGs were divided into the following two groups based on publication year: pre-2008 group (n = 22) and post-2008 group (n = 42). Additionally, we used the Mann-Whitney *U* test to compare the pre- and post-2008 groups in terms of differences in change in their scores by revision.

Two forms of CPGs exist; those for medical practitioners and those for patients. Our study included 22 CPGs for patients. The Mann-Whitney *U* test was used to compare CPGs for patients published until 2008 and those published since 2009.

A p-value of less than 0.05 was considered to indicate statistical significance. All analyses were performed using SPSS Statistics for Windows, version 20.0 (IBM Corp., Armonk, NY). All of the CPGs and AGREE I (including its manual) are published and accessible. No institutional review board approval was requested because the study used only open resources available in Japan.

## Results

### Characteristics of clinical practice guidelines

We identified 373 eligible CPGs. The number of published CPGs increased each year. Specifically, six CPGs were published in 2000, 17 in 2001–02, 32 in 2003–04, 47 in 2005–06, 44 in 2007–08, 66 in 2009–10, 68 in 2011–12, and 93 in 2013–14. Of these, 102 CPGs were revised, with 64 second editions and 38 third or later editions. Twenty-two CPGs for patients and 351 for medical practitioners were eligible for the present analysis (Table 1).

**Table 1:** Characteristics of evaluated clinical practice guidelines.

	Pre-2008					Post-2008			Total
	Before 2000	2001–02	2003–04	2005–06	2007–08	2009–10	2011–12	2013–14	
All	6	17	32	47	44	66	68	93	373
Edition									
1st edition	5	15	26	44	28	43	46	64	271
Revised edition	1	2	6	3	16	23	22	29	102
2nd edition	0	1	5	2	14	14	16	12	64
3rd edition or later	1	1	1	1	2	9	6	17	38
Version									
For patients	0	1	1	4	0	8	5	3	22
For medical practitioners	6	16	31	43	44	58	63	90	351

Respectively, the mean and median scores of the CPGs were as follows for the six domains: 89.6% and 93.0% for #1 (scope and purpose), 58.0% and 56.0% for #2 (stakeholder involvement), 58.1% and 62.0% for #3 (rigor of development), 73.4% and 75.0% for #4 (clarity of presentation), 39.8% and 37.0% for #5 (applicability), and 46.4% and 38.9% for #6 (editorial independence). Corresponding values for the overall score were 61.5% and 62.3%, respectively. Respectively, the mean and median scores for first-edition CPGs were as follows for the six domains: 88.9% and 92.6% for #1 (scope and purpose), 53.6% and 52.8% for #2 (stakeholder involvement), 56.2% and 60.0% for #3 (rigour of development), 71.8% and 75.0% for #4 (clarity of presentation), 38.1% and 37.0% for #5 (applicability), 44.4% and 33.3% for #6 (editorial independence). Corresponding values for the overall score were 59.3% and 60.4%, respectively.

### Time trend analysis of clinical practice guidelines

**Fig 1:**
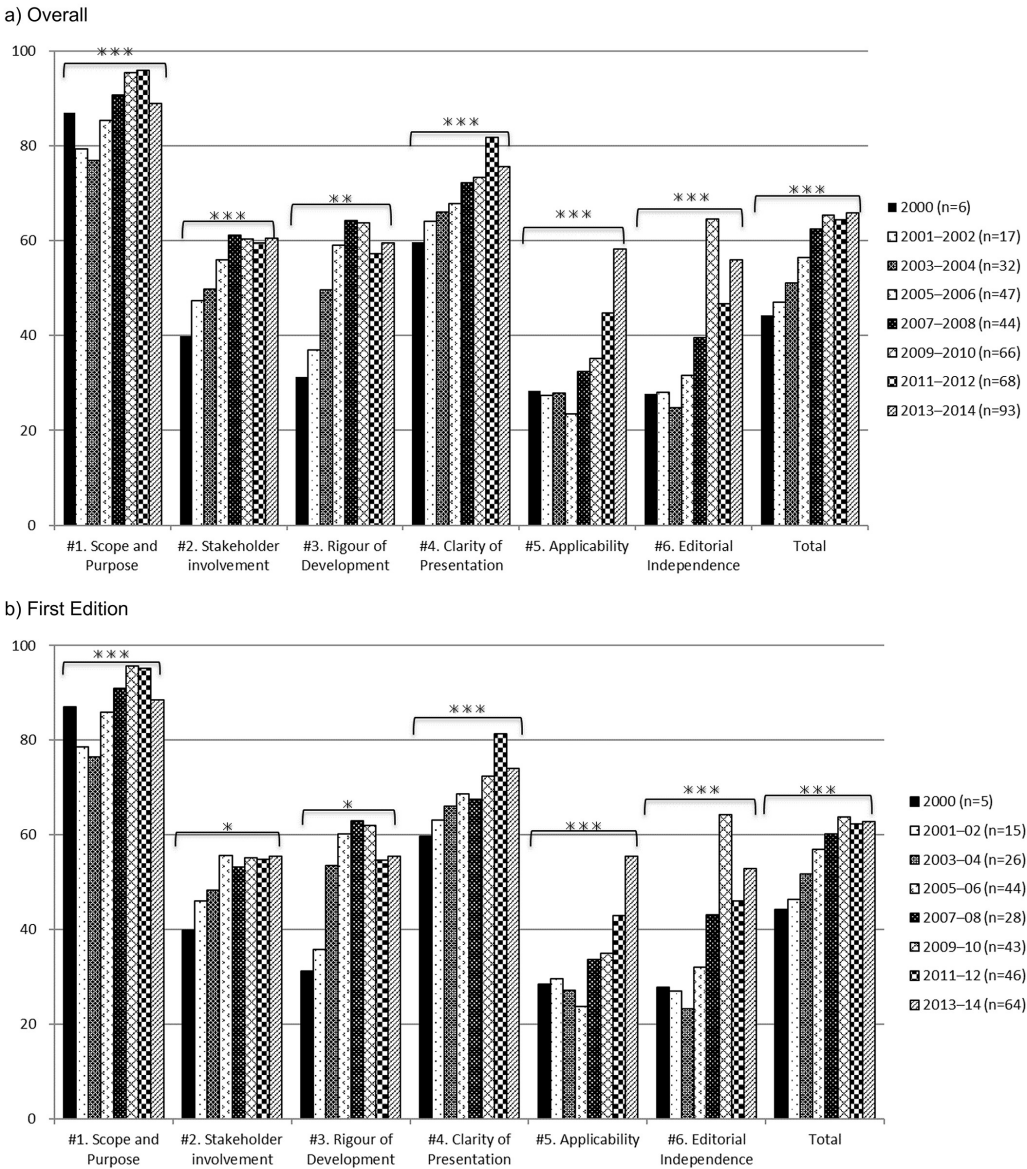
Time trend analysis of median scores of clinical practice guidelines. Test: Kruskal-Wallis test, *p < 0.05, **p < 0.01, ***p < 0.001.

Overall, the median scores for the CPGs improved each year on all domains. The same phenomenon was observed for first-edition CPGs (Fig 1).

### Comparison between first- and second-edition clinical practice guidelines

Respectively, the median scores of first- and second-edition CPGs were as follows for the six domains: 85.0% and 100.0% for #1 (scope and purpose) (p < 0.001), 50.0% and 69.0% for #2 (stakeholder involvement) (p < 0.001), 68.0% and 75.5% for #3 (rigor of development) (p = 0.001), 72.0% and 83.0% for #4 (clarity of presentation) (p < 0.001), 30.0% and 41.0% for #5 (applicability) (p < 0.001), 28.0% and 39.0% for #6 (editorial independence) (p < 0.001). Corresponding values for the overall score were 56.0% and 70.0% (p < 0.001), respectively. Evidently, the scores on all domains improved after revision (Table 2).

**Table 2:** Comparison of median scores of the first and second edition of clinical practice guidelines (n = 64).

	#1. Scope and Purpose	#2. Stakeholder Involvement	#3. Rigor of Development	#4. Clarity of Presentation	#5. Applicability	#6. Editorial Independence	Total
All paired CPGs (n = 64)							
1st ed.	85.0	50.0	68.0	72.0	30.0	28.0	56.0
2nd ed.	100.0	69.0	75.5	83.0	41.0	39.0	70.0
p value*	< 0.001	< 0.001	0.001	< 0.001	< 0.001	< 0.001	< 0.001
Pre-2008 (n = 22)							
1st ed.	87.0	44.0	40.5	72.0	26.0	28.0	51.5
2nd ed.	94.5	72.0	67.5	80.5	24.0	33.0	64.0
p value*	0.143	< 0.001	0.011	0.036	0.241	0.160	< 0.001
Post-2008 (n = 42)							
1st ed.	85.0	50.0	68.0	70.5	30.0	33.0	58.5
2nd ed. CPGs	100.0	69.0	78.5	83.0	52.0	50.0	72.5
p value*	< 0.001	< 0.001	0.023	< 0.001	< 0.001	< 0.001	< 0.001
Difference (post-pre)							
p value**	0.177	0.059	0.302	0.449	0.003	0.078	0.646

* Wilcoxon signed-rank test

** Mann-Whitney U test

Abbreviations: CPGs, clinical practice guidelines.

Based on publication year, second-edition CPGs (n = 64) were divided into pre- and post-2008 groups (n = 22 and 42, respectively). Compared with scores of first-edition CPGs, those of the pre-2008 group improved only in three domains (#2 stakeholder involvement, #3 rigor of development, and #4 clarity of presentation), whereas those of the post-2008 group improved in all domains. The percentage of difference between the post- and pre-2008 groups was 7.5% for #1 (scope and purpose), -9.0% for #2 (stakeholder involvement), -16.5% for #3 (rigour of development), 4.0% for #4 (clarity of presentation), 24.0% for #5 (applicability), and 12.0% for #6 (editorial independence). The percentage of difference in #5 was significantly higher in the post-2008 group as compared to that in the pre-2008 group (see Table 2).

### Comparison between pre- and post-2008 clinical practice guidelines for patients

Respectively, the median scores for pre- and post-2008 CPGs for patients were as follows for the six domains: 85.2% and 100.0% for #1 (scope and purpose) (p = 0.010), 69.4% and 66.7% for #2 (stakeholder involvement) (p = 1.000), 21.5% and 17.5% for #3 (rigour of development) (p = 0.407), 66.7% and 80.6% for #4 (clarity of presentation) (p = 0.178), 25.9% and 22.1% for #5 (applicability) (p = 0.590), and 27.8% and 30.4% for #6 (editorial independence) (p = 0.134). Corresponding values for the overall score were 45.4% and 48.6%, respectively (p = 0.178) (Table 3).

**Table 3:** Comparison between pre- and post-2008 groups in terms of median scores of clinical practice guidelines for patients (n = 22).

	#1. Scope and Purpose	#2. Stakeholder Involvement	#3. Rigor of Development	#4. Clarity of Presentation	#5. Applicability	#6. Editorial Independence	Total
Pre-2008 (n = 6)	85.2	69.4	21.5	66.7	25.9	27.8	45.4
Post-2008 (n = 16)	100.0	66.7	17.5	80.6	22.1	30.4	48.6
p*	0.010	1.000	0.407	0.178	0.590	0.134	0.178

*Mann-Whitney U test

## Discussion

### Time trend analysis

Similar to the results of a systematic review on quality of CPGs [17], our results showed that the number of published CPGs has been increasing, indicating an improvement in the quality of CPGs, as measured by the AGREE instrument. These trends were observed in the overall sample of CPGs (n = 373) and in first-edition CPGs (n = 271).

In 1999, Fukui et al. [18] published a list of diseases for which CPGs should be developed with priority. This list differed from a similar list developed in the United States [19], but it was one of the pioneer activities that quantified the social burden and room for improvement in the treatment of each disease. In 1999, MHLW began providing financial support for CPG development. Accordingly, between 1999 and 2006, MHLW established and financially supported 23 research groups for the development of CPGs. In 2001, the AGREE instrument was introduced in Japan. Guidelines for developing CPGs were also issued by JQ in 2007 (and revised in 2014), and CPG clearinghouses are currently being run by the JQ and Toho University Medical Media Center. Recently, the development of CPGs has become a customary activity of medical societies that engage many of their members. These changes in infrastructure might have contributed to the improvement in the quality of CPGs.

Detailed analyses of the six domains of the AGREE instrument may be indicators of how medical societies have responded to the changing demands of the Japanese society. The scores on Domain #1 (scope and purpose) were high (80–90% over the entire period), which indicates that CPGs described this domain sufficiently since the beginning. After 2007, a rapid increase was observed in scores on Domain #5 (applicability). In Japan, the process of approval of new drugs and devices was historically complex and lengthy. The simplification of this approval process became one of the three-year social regulatory reform goals of social concern in 2003 [20]. Additionally, it was included in the social reforms led by Prime Minister Abe, and the Pharmaceuticals and Medical Devices Agency simplified and shortened approval periods. These social events increased public interest in the application of new drugs and devices, which explains the improvement in scores on Domain #5 (applicability).

Scores on Domain #6 (editorial independence) skyrocketed during 2009–10. In Japan, abnormal behaviors in children accompanying the use of oseltamivir phosphate for influenza became a subject of discussion. MHLW established an expert panel to investigate the relationship between oseltamivir phosphate use and these behaviors. The panel ultimately failed to find any relationship, but several members of the panel were accused of receiving research funds from pharmaceutical companies that manufactured oseltamivir phosphate. In 2008, MHLW formulated “Guidelines on Management of Conflicts of Interest in Health Labor Science Research” [21], conflict of interest (COI) became a social concern, and several academic societies began to pay more attention to COI issues [22]. These factors may have influenced the increase in CPG quality pertaining to Domain #6.

Except for Domain #1 (scope and purpose) and #4 (clarity of presentation), whose median scores exceeded 75% in 2013–2014, this study identified room for quality improvement in all other domains. Our case study showed that provision of support for evaluating draft CPGs using the AGREE instrument and sending comments on such drafts during the development process led to improvements in all AGREE domains [23]. Therefore, to achieve improvements in the quality of CPGs, it may be beneficial to set steps for external review and amendment during the CPG development process.

### Differences before and after the revision of clinical practice guidelines

In a comparison of versions of CPGs before and after revision using a small sample, Bhatt et al. reported an increasing trend in median scores of revised versions in all domains, but this result was not statistically significant for four domains (#3 rigor of development, #4 clarity of presentation, #5 applicability, and #6 editorial independence) [24]. In the present study, a comparison of first- and second-edition CPGs (n = 64) showed that, after revision, scores improved significantly in all domains. Accumulation of experience and development of infrastructure for CPGs might explain this change.

Given that overall scores have been improving following revision, it is suggested that the quality of CPGs may be influenced by the circumstances in which they are developed and revised. The overall scores of second-edition CPGs were higher than those of first-edition CPGs. Additionally, significant improvement was observed in three domains in the pre-2008 group and in all domains in the post-2008 group. Compared with that of earlier CPGs, the degree of improvement of later CPGs was larger in Domain #5 (applicability; from -2.0% in pre-2008 to 22.0% in post-2008, p = 0.003). This improvement in Domain #5 may have been influenced by the increased social concern about COI.

### Comparison between clinical practice guidelines for patients published until 2008 and since 2009

A comparison between CPGs for patients showed that the score increased in only Domain #1 (Scope & Purpose). The Japanese society is aging rapidly, and the prevalence diseases related to quality of life and chronic disease is increasing [25]. Patients are themselves stakeholders, and in some cases, they have participated in the CPG development processes. The increasing awareness of the importance of preparing CPGs for patients may influence the improvement in scores on Domain #1. However, Japan has no organized patient associations, and most patient groups are generally small and fragmented, and they belong to one institution or are led by a few devoted individuals. Therefore, it may be difficult to establish solid partnerships with patients when developing CPGs. Strategies for promoting patient advocacy and encouraging patient participation in CPG development remain areas that need to be developed.

### Limitations of this study

Possible limitations of this study include (1) the comprehensive searching for Japanese EBM-based CPGs, (2) the limited reliability of CPG evaluation using the AGREE instrument, and (3) the influence of social events on changes in scores on specified domains.

Experienced librarians conducted a systematic review of CPGs and they hand-searched the literature on CPGs. Additionally, the CPGs were judged based on predefined criteria. Therefore, the method used in this study is considered highly reliable, and most, if not all, EBM-based CPGs were identified and included in the study. Therefore, the excluded CPGs may have had little influence on the results.

Three librarians rated each CPG independently, and these evaluators were experienced in CPG development. Interrater reliability was relatively high (the single and average measure intraclass correlation coefficients were 0.636 and 0.840, respectively), and standardized scores were calculated based on the three rating results. Therefore, the present results can be considered reliable.

This study aimed to investigate changes in CPG quality over time, with a focus on the infrastructure supporting CPG development, public interest in healthcare quality, and healthcare providers’ responses to this interest. Social experiments are difficult to reproduce, and causality is seldom demonstrated. However, a close look at social events can suggest their influence on the development and quality of CPGs.

We evaluated 373 CPGs published between 2000 and 2014 using the AGREE I instrument. Our results showed that the number of published CPGs increased during this period and the quality of CPGs has been improving consistently. Expanded infrastructure as well as the diffusion of experience and knowledge related to the development of CPGs among academic scholars and clinical practitioners could explain this improvement. Furthermore, CPG domain-level analyses suggested that healthcare providers have responded to changes in public interest in areas such as the approval lag for drugs and devices and COI issues. The results of our study suggested that the content of CPGs might reflect societal requirements for healthcare.

## Conclusion

The number of published CPGs has been increasing. Additionally, their quality, as measured with the AGREE instrument, has been improving. These changes seem to be influenced by improvements in social infrastructure, such as the publication of CPG development procedures, availability of CPG preparation methodology training, and increase in CPG-related skills.

## Supporting information

S1 Table List of clinical practice guidelines analyzed in the present study. (DOCX)

S2 Table Clinical practice guidelines’ standardized scores on each domain of AGREE (%). (DOCX)
