# Supplementary material for: Quality assessment of clinical practice guidelines using the AGREE instrument in Japan: A time trend analysis
Source: PLoS One. 2019 May 2;14(5):e0216346. doi: 10.1371/journal.pone.0216346 (PMC6497296; doi:10.1371/journal.pone.0216346)
Supplement: S1 Table — (DOCX) [file pone.0216346.s001.docx]

Table S1. List of clinical practice guidelines analyzed in the present study.

| No | Title | Development Groups | Publication year |
| --- | --- | --- | --- |
| 1 | Practical guideline for the management of allergic rhinitis in Japan <PG-MARJ> | Clinical Practice Guideline Development Group for the Management of Allergic Rhinitis | -2000 |
| 2 | Japanese Society of Hypertension Guidelines for the Management of Hypertension (JSH 2000) | The Japanese Society of Hypertension | -2000 |
| 3 | Clinical Practice Guideline for Diagnosis and Treatment of COPD | The Japanese Respiratory Society | -2000 |
| 4 | Clinical Practice Guideline for Diagnosis and Treatment of Respiratory failure | Research Committee on Respiratory failure of the Ministry of Health, Labour and Welfare of Japan | -2000 |
| 5 | Clinical Practice Guideline for Community-acquired Pneumonia | The Japanese Respiratory Society | -2000 |
| 6 | Clinical Practice Guideline for Prevention and Management of Asthma | Research Committee on Immunity and Allergy of the Ministry of Health, Labour and Welfare of Japan | -2000 |
| 7 | Clinical Practice Guideline for Treatment and Management of Severe Head injury | The Japan Society of Neurotraumatology | 2001 |
| 8 | Evidence-based Clinical Practice Guidelines for Acute Myocardial Infarction | Research Committee on Acute Myocardial Infarction and Ischemic Heart Disease of the Ministry of Health, Labour and Welfare of Japan | 2001 |
| 9 | Evidence-based Clinical Practice Guidelines for Management of Asthma | Research Committee on Asthma of the Ministry of Health, Labour and Welfare of Japan | 2001 |
| 10 | Japanese Gastric Cancer Treatment Guidelines 2001 (ver. 1) | Japanese Gastric Cancer Association | 2001 |
| 11 | Japanese Gastric Cancer Treatment Guidelines (for patient) | Japanese Gastric Cancer Association | 2001 |
| 12 | Evidence-based Clinical Practice Guidelines for Benign Prostatic Hyperplasia | Research Committee on Urological Disease of the Ministry of Health, Labour and Welfare of Japan | 2001 |
| 13 | Clinical Practice Guideline for Management of Eating Disorder | Research Committee on Eating Disorder of the Ministry of Health, Labour and Welfare of Japan | 2002 |
| 14 | Japan Atherosclerosis Society (JAS) Guidelines for Diagnosis and Treatment of Atherosclerotic Cardiovascular Diseases | Japan Atherosclerosis Society | 2002 |
| 15 | Clinical Practice Guideline for Management of Dementia | Japanese Society of Neurology | 2002 |
| 16 | Practical Guideline for the Management of Allergic Rhinitis in Japan <PG-MARJ> | Clinical Practice Guideline Development Group for the Management of Allergic Rhinitis | 2002 |
| 17 | Clinical Practice Guideline for Hospital-acquired Pneumonia in Adult | The Japanese Respiratory Society | 2002 |
| 18 | Japanese Pediatric Guideline for the Treatment and Management of Asthma | Japanese Society of Pediatric Allergy and Clinical Immunology | 2002 |
| 19 | Clinical Practice Guideline for Management of Sleep Disorder | Japanese Study Group on Diagnosis and Treatment Guideline of a Sleep Disorder | 2002 |
| 20 | Clinical Practice Guideline for Mental Trauma | Research Committee on Traumatic Stress-related Disorders of the Ministry of Health, Labour and Welfare of Japan | 2002 |
| 21 | Evidence-based Clinical Practice Guidelines for Management of Cancer Pain | Japanese Society for Palliative Medicine | 2002 |
| 22 | Clinical Practice Guideline for Management of Esophagus Cancer | The Japanese Society for Esophageal Diseases | 2002 |
| 23 | Clinical Practice Guideline for Urolithiasis | The Japanese Urological Association, Japanese Society of Endourology and ESWL, Japanese Society on Urolithiasis Research | 2002 |
| 24 | Evidence-based Clinical Practice Guidelines for Acute Pancreatitis | Clinical Practice Guideline Development Group for the Acute Pancreatitis | 2003 |
| 25 | Clinical Practice Guideline for Urolithiasis Respiratory Tract Infection in Adult | The Japanese Respiratory Society | 2003 |
| 26 | Evidence-based Clinical Practice Guidelines for Gastric Ulcer | Research Committee on Gastric Ulcer of the Ministry of Health, Labour and Welfare of Japan | 2003 |
| 27 | Clinical Practice Guideline for Prevention and Management of Asthma | Research Committee on Immunity and Allergy of the Ministry of Health, Labour and Welfare of Japan | 2003 |
| 28 | Evidence-based Clinical Practice Guidelines for Parkinson's Disease | Japanese Society of Neurology | 2003 |
| 29 | Clinical Practice Guideline for the Diagnosis and Treatment of Alcohol and Drug-related Disorder | Japanese Study Group on Diagnosis and Management of Alcohol and Drug-related Disorder | 2003 |
| 30 | Guidelines for Management of Deep-seated Mycoses | The Japanese Society for Medical Mycology | 2003 |
| 31 | Clinical Practice Guideline for Diagnosis and Management of Attention Deficit-Hyperactivity Disorder (AD/HD) | Japanese Study Group on Diagnosis and Management of AD/HD | 2003 |
| 32 | Evidence-based Clinical Practice Guidelines for Lung Cancer | Research Committee on Lung Cancer of the Ministry of Health, Labour and Welfare of Japan | 2003 |
| 33 | Clinical Practice Guideline for Prevention of Pulmonary Thromboembolism and Deep Vein Thrombosis | Clinical Practice Guideline Development Group for the Pulmonary Thromboembolism and Deep Vein Thrombosis | 2004 |
| 34 | Clinical Practice Guideline for Hyperlipemia | Japan Atherosclerosis Society | 2004 |
| 35 | Evidence-based Practice Guideline for the Treatment of Diabetes in Japan | The Japan Diabetes Society | 2004 |
| 36 | Guidelines for the Management of Hypertension (JSH 2004) | The Japanese Society of Hypertension | 2004 |
| 37 | Clinical Practice Guideline for Multiple Myeloma | Japan Myeloma Study Group | 2004 |
| 38 | Clinical Practice Guideline for Diagnosis and Treatment of COPD | The Japanese Respiratory Society | 2004 |
| 39 | Idiopathic Interstitial Pneumonias : Diagnosis and Treatment | The Japanese Respiratory Society | 2004 |
| 40 | Evidence-based Clinical Practice Guidelines for Management of Asthma | Research Committee on Database of Clinical Practice Guideline of the Ministry of Health, Labour and Welfare of Japan | 2004 |
| 41 | Evidence-based Clinical Practice Guidelines for Perinatal Domestic Violence | Research Group for Women's Centered Care, St. Luke's Nursing University | 2004 |
| 42 | Clinical Practice Guideline for Asthma (for medical staff) | Japanese Society of Pediatric Allergy and Clinical Immunology | 2004 |
| 43 | Japanese Pediatric Guideline for the Treatment and Management of Asthma | Japanese Society of Pediatric Allergy and Clinical Immunology | 2004 |
| 44 | Evidence-based Clinical Practice Guidelines for Diagnosis of Rheumatoid Arthritis | Research Committee on Rheumatoid Arthritis of the Ministry of Health, Labour and Welfare of Japan | 2004 |
| 45 | Japanese Guidelines for the Management of Stroke (2004) | The Joint Committee on Guidelines for the Management of Stroke | 2004 |
| 46 | Evidence-based Clinical Practice Guidelines for Subarachnoid Hemorrhage | Japanese Society on Surgery for Cerebral Stroke | 2004 |
| 47 | Guidelines for Treatment of Autoimmune Diseases of the Nervous System | Clinical Practice Guideline Development Group for the Autoimmune Diseases of the Nervous System | 2004 |
| 48 | Clinical Practice Guideline for Idiopathic Normal Pressure Hydrocephalus | Japan Society of Normal Pressure Hydrocephalus | 2004 |
| 49 | Clinical Practice Guideline for Mood Disorders | The Committee on Psychiatry | 2004 |
| 50 | Japanese Gastric Cancer Treatment Guidelines 2004 (ver. 2) | Japanese Gastric Cancer Association | 2004 |
| 51 | Japanese Gastric Cancer Treatment Guidelines (for patient) | Japanese Gastric Cancer Association | 2004 |
| 52 | Evidence-based Clinical Practice Guidelines for Breast Cancer 1, Pharmacotherapy | Japanese Breast Cancer Society | 2004 |
| 53 | Clinical Practice Guideline for Ovarian Cancer | The Japan Society of Gynecologic Oncology | 2004 |
| 54 | Evidence-based Clinical Practice Guidelines for Incontinence | Research Committee on Urological Disease of the Ministry of Health, Labour and Welfare of Japan | 2004 |
| 55 | Guidelines for the Management of Respiratory Infectious Diseases in Children in Japan | Clinical Practice Guideline Development Group for the Management of Respiratory Infectious Diseases in Children | 2004 |
| 56 | Clinical Practice Guideline for Mixed Connective Tissue Disease | Research Committee on Mixed Connective Tissue Disease of the Ministry of Health, Labour and Welfare of Japan | 2005 |
| 57 | Clinical Practical Guideline for Acute Lung Injury and Acute Respiratory Distress Syndrome | The Japanese Respiratory Society | 2005 |
| 58 | Evidence-based Clinical Practice Guidelines for Acute Cholangitis and Acute Cholecystitis | Clinical Practice Guideline Development Group for the Acute cholangitis | 2005 |
| 59 | Guideline for Diagnosis & Treatment of Adult SAS | Japanese Study Group on Sleep Disordered Breathing | 2005 |
| 60 | Japanese Pediatric Guideline for Food Allergy | Japanese Society of Pediatric Allergy and Clinical Immunology | 2005 |
| 61 | JSCCR Guidelines for the Treatment of Colorectal Cancer | Japanese Society for Cancer of the Colon and Rectum | 2005 |
| 62 | Evidence-based Clinical Practice Guidelines for Pancreatic Cancer | Japan Pancreas Society | 2005 |
| 63 | Evidence-based Clinical Practice Guidelines for Liver Cancer | Research Committee on Liver Cancer of the Ministry of Health, Labour and Welfare of Japan | 2005 |
| 64 | Evidence-based Clinical Practice Guidelines for Breast Cancer 2, Surgical practice | Japanese Breast Cancer Society | 2005 |
| 65 | Evidence-based Clinical Practice Guidelines for Breast Cancer 3, Radiation Therapy | Japanese Breast Cancer Society | 2005 |
| 66 | Evidence-based Clinical Practice Guidelines for Breast Cancer 4, Examination and Diagnosis | Japanese Breast Cancer Society | 2005 |
| 67 | Evidence-based Clinical Practice Guidelines for Breast Cancer 5, Epidemiology and Prevention | Japanese Breast Cancer Society | 2005 |
| 68 | Evidence-based Clinical Practice Guidelines for Lung Cancer | The Japan Lung Cancer Society | 2005 |
| 69 | Clinical Practice Guideline for Femoral Neck Fracture and Intertrochanteric Femoral Fracture | The Japanese Orthopaedic Association, Research Committee on Femoral Neck Fracture of the Ministry of Health, Labour and Welfare of Japan | 2005 |
| 70 | Clinical Practice Guideline for Ossification of Posterior Longitudinal Ligament of the Cervical Spine | The Japanese Orthopaedic Association, Research Committee on Spinal Ligament of the Ministry of Health, Labour and Welfare of Japan | 2005 |
| 71 | Clinical Practice Guideline for Cervical Spondylotic Myelopathy | The Japanese Orthopaedic Association | 2005 |
| 72 | Clinical Practice Guideline for Diagnosis of Soft-tissue Tumor | The Japanese Orthopaedic Association | 2005 |
| 73 | Clinical Practice Guideline for Lumbar Disc Herniation | The Japanese Orthopaedic Association, Research group for Lumbar Disc Herniation of the Ministry of Health, Labour and Welfare of Japan | 2005 |
| 74 | Guideline for Local Treatment of Pressure Ulcers | Japanese Society of Pressure Ulcers | 2005 |
| 75 | Clinical Practice Guideline for Overactive Bladder | The Japanese Continence Society | 2005 |
| 76 | Clinical Practice Guideline for Anterior Cruciate Ligament Injury | The Japanese Orthopaedic Association | 2006 |
| 77 | Clinical Practice Guideline for Management of Obesity | Japan Society for the Study of Obesity | 2006 |
| 78 | Clinical Practice Guideline for Assessment and Management of Drug-induced Lung Disorder | The Japanese Respiratory Society | 2006 |
| 79 | JSCCR Guidelines for the Treatment of Colorectal Cancer (for patient) | Japanese Society for Cancer of the Colon and Rectum | 2006 |
| 80 | Guideline for Obstetrics and Gynecology Practice Based on Updated Evidence | Yuji Taketani [editor] | 2006 |
| 81 | Clinical Practical Guideline for Rheumatoid Arthritis (for Patient) | Japan Rheumatism Foundation | 2006 |
| 82 | Clinical Practice Guideline for Parenteral & Enteral Nutrition | Japanese Society for Parenteral & Enteral Nutrition | 2006 |
| 83 | Clinical Practice Guideline for Lateral Epicondylitis | The Japanese Orthopaedic Association | 2006 |
| 84 | Guideline for the Treatment of Graves' Disease with Antithyroid Drug in Japan | Japan Thyroid Association | 2006 |
| 85 | Clinical Practice Guideline for　Chronic Headache | The Japanese Headache Society | 2006 |
| 86 | Evidence-based Clinical Practice Guidelines for Pancreatic Cancer | Japan Pancreas Society | 2006 |
| 87 | Clinical Practice Guideline for Noninvasive Positive Pressure Ventilation Therapy | The Japanese Respiratory Society | 2006 |
| 88 | International Consensus Guidelines for Management of Intraductal Papillary Mucinous Neoplasms and Mucinous Cystic Neoplasms of the Pancreas: Japanese version | International Association of Pancreatology | 2006 |
| 89 | A Guideline for the Diagnosis and Treatment of Psychosomatic Diseases | Clinical Practice Guideline Development Group for the Diagnosis and Management of Psychosomatic Diseases | 2006 |
| 90 | Clinical Practice Guideline for Prevention of Surgical Site Infection (bone and joint) | The Japanese Orthopaedic Association | 2006 |
| 91 | Clinical Practice Guideline for Prostate Cancer | The Japanese Urological Association | 2006 |
| 92 | Guidelines for Acute Otitis Media in Children | Japan Otological Society, Japan Society for Pediatric ORL, Japan Society for Infectious Diseases in Otolaryngology | 2006 |
| 93 | Clinical Practice Guideline for　Uterine Cancer | The Japan Society of Gynecologic Oncology | 2006 |
| 94 | Clinical Practice Guideline for Prevention and Management of Osteoporosis | Japan Osteoporosis Society, The Japanese Society for Bone and Mineral Research, Japan Osteoporosis Foundation, Research Committee on Osteoporosis of the Ministry of Health, Labour and Welfare of Japan | 2006 |
| 95 | Guidelines for the Management of Atopic Dermatitis | Japanese Society of Allergology | 2006 |
| 96 | Clinical Practice Guideline for Treatment and Management of Severe Head injury | Clinical Practice Guideline Development Group for the Treatment and Management of Severe Head Injury | 2006 |
| 97 | Evidence-based Clinical Practice Guidelines for Breast Cancer (for Patient) | Japanese Breast Cancer Society | 2006 |
| 98 | Clinical Practice Guideline for Emergency Resuscitation | Clinical Practice Guideline Development Group for the Resuscitation | 2006 |
| 99 | Clinical Practice Guideline for Oxygen Therapy | The Japanese Respiratory Society, Japan Society for Respiratory Care | 2006 |
| 100 | Clinical Practice Guideline for Prevention and Management of Asthma (JSL2006) | Research Committee on Immunity and Allergy of the Ministry of Health, Labour and Welfare of Japan | 2006 |
| 101 | Clinical Practice Guideline for Treatment and Management of Severe Head Injury | Clinical Practice Guideline Development Group for the Treatment and Management of Severe Head Injury | 2006 |
| 102 | Guidelines for the Management of Ulcerative Colitis in Japan ―Developed through Integration of Evidence and Consensus among Experts | Research Committee on Intractable Inflammatory Bowel Disorders of the Ministry of Health, Labour and Welfare of Japan | 2006 |
| 103 | Evidence-based Clinical Practice Guidelines for Gastric Ulcer | Research Committee on Gastric Ulcer of the Ministry of Health, Labour and Welfare of Japan | 2007 |
| 104 | Clinical Practice Guideline for　rupture of Achilles Tendon | The Japanese Orthopaedic Association | 2007 |
| 105 | Evidence-based Clinical Practice Guidelines for Acute Pancreatitis | Clinical Practice Guideline Development Group for the Acute Pancreatitis | 2007 |
| 106 | Evidence-based Practice Guideline for the Treatment of Diabetes in Japan | The Japan Diabetes Society | 2007 |
| 107 | Evidence-based Clinical Practice Guidelines for Breast Cancer 1, Pharmacotherapy | Japanese Breast Cancer Society | 2007 |
| 108 | Evidence-based Clinical Practice Guidelines for Skin Cancer | Japanese Skin Cancer Society | 2007 |
| 109 | Clinical Practice Guideline for Late Onset Hypogonadism Syndrome | The Japanese Urological Association, Japanese Society of Men's Health | 2007 |
| 110 | Clinical Practice Guideline for Interstitial Cystitis | Society of Interstitial Cystitis of Japan | 2007 |
| 111 | Clinical Practice Guideline for Diagnostic Imaging of Acute Cerebral Infarction | Clinical Practice Guideline Development Group for the ASIST-Japan | 2007 |
| 112 | Clinical Practice Guideline for Bacterial Meningitis | Japanese Society of Neurological Therapeutics, Japanese Society of Neurology, Japanese Society for Neuroinfectious Diseases | 2007 |
| 113 | Clinical Practice Guideline for Cervical Cancer | The Japan Society of Gynecologic Oncology | 2007 |
| 114 | Clinical Practice Guideline for Diagnosis and Management of Periodontal Disease | Japanese Society of Periodontology | 2007 |
| 115 | Guidelines for the Management of Respiratory Infectious Diseases in Children in Japan | Clinical Practice Guideline Development Group for the Management of Respiratory Infectious Diseases in Children | 2007 |
| 116 | Clinical Practice Guideline for Infantile Leukemia and Lymphoma | Japanese Society of Pediatric Hematology | 2007 |
| 117 | Guidelines for Diagnosis and Treatment of Carcinoma of the Esophagus | The Japan Esophageal Society | 2007 |
| 118 | Clinical Practice Guideline for Renal Cancer | The Japanese Urological Association | 2007 |
| 119 | The JRS Guidelines for the Management of Community-acquired Pneumonia in Adults | The Japanese Respiratory Society | 2007 |
| 120 | Clinical Practice Guideline for Resin-bonded Fixed Partial Denture | Japan Prosthodontic Society | 2007 |
| 121 | Japan Atherosclerosis Society (JAS) Guidelines for Prevention of Atherosclerotic Cardiovascular Diseases | Japan Atherosclerosis Society | 2007 |
| 122 | Clinical Practice Guideline for Cerebrospinal Fluid Hypovolemia (CSF) | Japan CSF Hypovolemia Society | 2007 |
| 123 | Clinical Practice Guideline for Sinusitis | The Japanese Rhinologic Society | 2007 |
| 124 | Clinical Practice Guideline for Plate Denture Prosthesis | Japan Prosthodontic Society | 2007 |
| 125 | Clinical Practice Guideline for Ovarian Cancer | The Japan Society of Gynecologic Oncology | 2007 |
| 126 | JSSM Guidelines for Erectile Dysfunction | The Japanese Society for Sexual Medicine | 2007 |
| 127 | Clinical Practice Guidelines for　Dysphagia in Otorhinolaryngology Outpatient Department | The Oto-Rhino-Laryngological Society of Japan | 2008 |
| 128 | Clinical Practice Guideline for　 Hallux Valgus | The Japanese Orthopaedic Association | 2008 |
| 129 | Nursing Guideline for Outpatient Cancer Chemotherapy | Working group for Outpatient cancer chemotherapy nursing, St. Luke's Nursing University | 2008 |
| 130 | Clinical Practice Guideline for GIST | Japan Society of Clinical Oncology  , Japanese Gastric Cancer Association, Japanese Study Group on GIST | 2008 |
| 131 | Practical Guideline for the Management of Allergic Rhinitis in Japan <PG-MARJ> | Clinical Practice Guideline Development Group for the Management of Allergic Rhinitis | 2008 |
| 132 | Clinical Practice Guideline for Coxarthrosis | The Japanese Orthopaedic Association | 2008 |
| 133 | Clinical Practice Guideline for Urinary Organ Laparoscopic Surgery | Japanese Society of Endourology and ESWL, Japan Society for Endoscopic Surgery | 2008 |
| 134 | Evidence-based Clinical Practice Guidelines for Breast Cancer 2, Surgical practice | Japanese Breast Cancer Society | 2008 |
| 135 | Evidence-based Clinical Practice Guidelines for Breast Cancer 3, Radiation therapy | Japanese Breast Cancer Society | 2008 |
| 136 | Evidence-based Clinical Practice Guidelines for Breast Cancer 4, Examination and Diagnosis | Japanese Breast Cancer Society | 2008 |
| 137 | Evidence-based Clinical Practice Guidelines for Breast Cancer 5, Epidemiology and Prevention | Japanese Breast Cancer Society | 2008 |
| 138 | Clinical Practice Guideline for Venous Thrombosis | The Japanese Orthopaedic Association | 2008 |
| 139 | Clinical Practice Guideline for Brain Dock | The Japan Brain Dock Society | 2008 |
| 140 | Guideline for Breast Ultrasound : Management and Diagnosis | The Japan Association of Breast and Thyroid Sonology | 2008 |
| 141 | The JRS Guidelines for the Management of Hospital-acquired Pneumonia in Adults | The Japanese Respiratory Society | 2008 |
| 142 | Japanese Pediatric Guideline for the Treatment and Management of Asthma | Japanese Society of Pediatric Allergy and Clinical Immunology | 2008 |
| 143 | Clinical Practice Guideline for Multiple Myeloma | Japan Myeloma Study Group | 2008 |
| 144 | Clinical Practice Guideline for Schizophrenia | The Committee on Psychiatry | 2008 |
| 145 | Screening Guideline for Prostate Cancer | The Japanese Urological Association | 2008 |
| 146 | Guidelines for the Management of Ulcerative Colitis in Japan ―Developed through Integration of Evidence and Consensus among Experts IBD Research | Research Committee on Intractable Inflammatory Bowel Disorders of the Ministry of Health, Labour and Welfare of Japan | 2008 |
| 147 | Guidelines for the Management of Hypertension (JSH 2009) | The Japanese Society of Hypertension | 2009 |
| 148 | Clinical Practice Guideline for　Lymphedema | Clinical Practice Guideline Development Group for the Lymphedema | 2009 |
| 149 | Clinical Practice Guideline for Prevention and Management of Asthma | Clinical Practice Guideline Development Group for the Prevention and Management of Asthma | 2009 |
| 150 | Clinical Practice Guideline for Diagnosis and Treatment of COPD | The Japanese Respiratory Society | 2009 |
| 151 | Evidence-based Clinical Practice Guidelines for Peptic Ulcer | The Japanese Society of Gastroenterology | 2009 |
| 152 | Clinical Practice Guideline for Diagnosis and Management of H. pylori Infection | The Japanese Society for Helicobacter Research | 2009 |
| 153 | Evidence-based Clinical Practice Guidelines for GERD | The Japanese Society of Gastroenterology | 2009 |
| 154 | Clinical Practice Guideline for Acute Pancreatitis | Clinical Practice Guideline Development Group for the Acute Pancreatitis | 2009 |
| 155 | Clinical Practice Guideline for Cholelithiasis | The Japanese Society of Gastroenterology | 2009 |
| 156 | Clinical Practice Guideline for Chronic Pancreatitis | The Japanese Society of Gastroenterology | 2009 |
| 157 | Clinical Practice Guideline for Tuberculosis | The Japanese Society for Tuberculosis | 2009 |
| 158 | Clinical Practice Guideline for Burn Injuries | Japanese Society for Burn Injuries | 2009 |
| 159 | JSCCR Guidelines for the Treatment of Colorectal Cancer | Japanese Society for Cancer of the Colon and Rectum | 2009 |
| 160 | JSCCR Guidelines for the Treatment of Colorectal Cancer (for Patient) | Japanese Society for Cancer of the Colon and Rectum | 2009 |
| 161 | Evidence-based Clinical Practice Guidelines for Liver Cancer | Research Committee on Liver Cancer of the Ministry of Health, Labour and Welfare of Japan | 2009 |
| 162 | Evidence-based Clinical Practice Guidelines for Pancreatic Cancer | Japan Pancreas Society | 2009 |
| 163 | Clinical Practice Guideline for Bladder Cancer | The Japanese Urological Association | 2009 |
| 164 | Clinical Practice Guideline for Testicular Cancer | The Japanese Urological Association | 2009 |
| 165 | Clinical Practice Guideline for Uterine Cancer | The Japan Society of Gynecologic Oncology | 2009 |
| 166 | Clinical Practice Guideline for Head and Neck Cancer | Japan Society for Head and Neck Cancer | 2009 |
| 167 | Evidence-based Clinical Practice Guidelines for Oral Cancer | Japanese Society of Oral Oncology, Japanese Society of Oral and Maxillofacial Surgeons | 2009 |
| 168 | Clinical Practice Guideline for Rehabilitation of Cerebral Palsy | Japanese Association of Rehabilitation Medicine | 2009 |
| 169 | Guidelines for the Management of Atopic Dermatitis | Japanese Society of Allergology | 2009 |
| 170 | Guideline for Prevention and Management of Pressure Ulcers | Japanese Society of Pressure Ulcers | 2009 |
| 171 | Evidence-based Practice Guideline for the Treatment of CKD | Japanese Society of Nephrology | 2009 |
| 172 | Clinical Guideline for Nocturia | The Japanese Continence Society | 2009 |
| 173 | Hormone Replacement Therapy Guideline | Japan Society of Obstetrics and Gynecology, Japan Society for Menopause and Women's Health | 2009 |
| 174 | Guideline 2009 for Care and Treatment of Hypertension in Pregnancy (PIH) | Japan Society for the Study of Hypertension in Pregnancy | 2009 |
| 175 | Guidelines for Acute Otitis Media in Children | Japan Otological Society, Japan Society for Pediatric ORL, Japan Society for Infectious Diseases in Otolaryngology | 2009 |
| 176 | Clinical Practice Guideline for Periodontal Treatment of Diabetic Mellitus Patient | Japanese Society of Periodontology | 2009 |
| 177 | Evidence-based Clinical Practice Guidelines for Breast Cancer (for patient) | Japanese Breast Cancer Society | 2009 |
| 178 | Practical Guideline for the Management of Allergic Rhinitis in Japan <PG-MARJ> | Clinical Practice Guideline Development Group for the Management of Allergic Rhinitis | 2009 |
| 179 | Guidelines for the Management of Stroke in Japan | The Joint Committee on Guidelines for the Management of Stroke | 2009 |
| 180 | Clinical Practical Guideline for Cervical Vertebra Ossification of Posterior Longitudinal Ligament (for patient) | The Japanese Orthopaedic Association, Research Committee on Spinal Ligament of the Ministry of Health, Labour and Welfare of Japan | 2009 |
| 181 | Clinical Practical Guideline for Amyloidosis | Research Committee on Amyloidosis of the Ministry of Health, Labour and Welfare of Japan | 2010 |
| 182 | Evidence-based Practice Guideline for the Treatment of Diabetes in Japan | The Japan Diabetes Society | 2010 |
| 183 | Clinical Practical Guideline for Hyperuricemia and Gout | Japanese Society of Gout and Nucleic Acid Metabolism | 2010 |
| 184 | Clinical Practical Guideline for Cholesterol | Japan Society for Lipid Nutrition | 2010 |
| 185 | Clinical Practical Guideline for Acute Lung Injury and Acute Respiratory Distress Syndrome | The Japanese Respiratory Society | 2010 |
| 186 | Evidence-based Clinical Practice Guidelines for Peptic Ulcer (for patient) | The Japanese Society of Gastroenterology | 2010 |
| 187 | Evidence-based Clinical Practice Guidelines for GERD (for Patient) | The Japanese Society of Gastroenterology | 2010 |
| 188 | Clinical Practical Guideline for Crohn Disease | The Japanese Society of Gastroenterology | 2010 |
| 189 | Clinical Practical Guideline for Primary Biliary Cirrhosis (PBC) | Research Committee on Intractable Hepatic and Biliary Tract Disease of the Ministry of Health, Labour and Welfare of Japan | 2010 |
| 190 | Clinical Practice Guideline for Chronic pancreatitis (for Patient and Family) | The Japanese Society of Gastroenterology | 2010 |
| 191 | Evidence-based Clinical Practice Guidelines for Liver Cirrhosis | The Japanese Society of Gastroenterology | 2010 |
| 192 | Clinical Practice Guideline for Cholelithiasis (for Patient and Family) | The Japanese Society of Gastroenterology | 2010 |
| 193 | Clinical Practice Guideline for Fibromyalgia | Japan College of Fibromyalgia Investigation | 2010 |
| 194 | Clinical Practice Guideline for Management of Epilepsy | Japanese Society of Neurology | 2010 |
| 195 | Clinical Practice Guideline for Management of Dementing Disorder | Japanese Society of Neurology | 2010 |
| 196 | Clinical Practice Guideline for Management of Multiple Sclerosis | Japanese Society of Neurology, The Japanese Society for Neuroimmunology, Japanese Society of Neurological Therapeutics | 2010 |
| 197 | Clinical Practice Guideline for Mood Disorders | The Committee on Psychiatry | 2010 |
| 198 | Clinical Practice Guideline for Management of Pain Clinic | Japan Society of Pain Clinicians | 2010 |
| 199 | Clinical Practice Guideline for Pharmacotherapy of Cancer Pain | Japanese Society for Palliative Medicine | 2010 |
| 200 | Clinical Practice Guideline on Sedation for Palliative Care | Japanese Society for Palliative Medicine | 2010 |
| 201 | Clinical Practice Guideline for Carcinoma of Unknown Primary | Japanese Society of Medical Oncology | 2010 |
| 202 | Clinical Practice Guideline for Proper Use of Antiemetic | Japan Society of Clinical Oncology | 2010 |
| 203 | Evidence-based Clinical Practice Guidelines for Breast Cancer 1, Pharmacotherapy | Japanese Breast Cancer Society | 2010 |
| 204 | Clinical Practice Guideline for GIST | Japan Society of Clinical Oncology, Japanese Gastric Cancer Association, Japanese Study Group on GIST | 2010 |
| 205 | Japanese Gastric Cancer Treatment Guidelines 2010 (ver. 3) | Japanese Gastric Cancer Association | 2010 |
| 206 | JSCCR Guidelines for the Treatment of Colorectal Cancer | Japanese Society for Cancer of the Colon and Rectum | 2010 |
| 207 | Clinical Practice Guideline for Thyroid Tumor | Japan Association of Endocrine Surgery, Japan Thyroid Association | 2010 |
| 208 | Clinical Practice Guideline for Ovarian Cancer | The Japan Society of Gynecologic Oncology | 2010 |
| 209 | Evidence-based Clinical Practice Guidelines for malignant skin tumor Ⅱ | The Japanese Dermatological Association | 2010 |
| 210 | Clinical Practice Guideline for Cervical Spondylotic Myelopathy (for patient) | The Japanese Orthopaedic Association | 2010 |
| 211 | Clinical Practice Guideline for Diagnosis and Management of Fetal Hydrocephalus | Clinical Practice Guideline Development Group for the Fetal Hydrocephalus | 2010 |
| 212 | Clinical Practice Guideline for Management of Dental Caries | The Japanese Society of Conservative Dentistry | 2010 |
| 213 | Clinical Practice Guideline for Resuscitation | Japan Resuscitation Council, Japan Emergency Care Foundation | 2011 |
| 214 | Clinical Practice Guideline for Mixed Connective Tissue Disease | Research Committee for Mixed Connective Tissue Disease of the Ministry of Health, Labour and Welfare, Japan | 2011 |
| 215 | Clinical Practice Guideline for ANCA-associated Vasculitis | Research Committee for ANCA-associated vasculitis, Intractable Vasculitis and Progressive Renal Disorder of the Ministry of Health, Labour and Welfare, Japan | 2011 |
| 216 | Clinical Practice Guideline for Healthcare Associated Pneumonia | The Japanese Respiratory Society | 2011 |
| 217 | Clinical Practice Guideline for Crohn Disease (for patient and family) | The Japanese Society of Gastroenterology | 2011 |
| 218 | Clinical Practice Guideline for Crohn Disease | Research Committee on Intractable Inflammatory Bowel Disorders of the Ministry of Health, Labour and Welfare of Japan, The Japanese Society of Gastroenterology | 2011 |
| 219 | Evidence-based Clinical Practice Guidelines for Liver Cirrhosis (for patient and family) | The Japanese Society of Gastroenterology | 2011 |
| 220 | Clinical Practice Guideline for Primary Biliary Cirrhosis | Research Committee on Intractable Hepatic and Biliary Tract Disease of the Ministry of Health, Labour and Welfare of Japan | 2011 |
| 221 | Guidelines for the Treatment of Graves' Disease in Japan | Japan Thyroid Association | 2011 |
| 222 | Clinical Practice Guideline for Management of Rheumatoid Arthritis | Japan College of Rheumatology | 2011 |
| 223 | Clinical Practice Guideline for Home Music Therapy of Amyotrophic Lateral Sclerosis (ALS) | Research Committee on QOL of Specified Disease Patient of the Ministry of Health, Labour and Welfare of Japan | 2011 |
| 224 | Clinical Practice Guideline for Diagnosis and Management of Fetal Hydrocephalus | The Japanese Society of NPH | 2011 |
| 225 | Clinical Practice Guideline for Facial Palsy; Bell's palsy and Hunt syndrome | Japan Society of Facial Nerve Research | 2011 |
| 226 | Evidence-based Clinical Practice Guidelines for Parkinson's Disease | Japanese Society of Neurology | 2011 |
| 227 | Clinical Practice Guideline for Management of Diabetes in Children and Puberty | The Japan Diabetes Society, The Japanese Society for Pediatric Endocrinology | 2011 |
| 228 | Clinical Practice Guideline for Pediatric Oncology | Japanese Society of Pediatric Oncology | 2011 |
| 229 | Clinical Practice Guideline for Pharmacotherapy of Neuropathic Pain | Japan Society of Pain Clinicians | 2011 |
| 230 | Clinical Practice Guideline for  Internal Medicine and Pediatric Complication after Kidney Transplantation | Japanese Society for Clinical Renal Transplantation | 2011 |
| 231 | Clinical Guidelines for Gastrointestinal Symptoms in Cancer Patients | Japanese Society for Palliative Medicine | 2011 |
| 232 | Clinical Guidelines for Respiratory Symptoms In Cancer Patients | Japanese Society for Palliative Medicine | 2011 |
| 233 | Evidence-based Clinical Practice Guideline for Breast Cancer 1, Treatment | Japanese Breast Cancer Society | 2011 |
| 234 | Evidence-based Clinical Practice Guidelines for Breast Cancer 2, Epidemiology and Diagnosis | Japanese Breast Cancer Society | 2011 |
| 235 | Clinical Practice Guideline for Cervical Cancer, Uterine Cancer and Ovarian Cancer (for patient) | The Japan Society of Gynecologic Oncology | 2011 |
| 236 | Clinical Practice Guideline for Ossification of Posterior Longitudinal Ligament of the Cervical Spine | The Japanese Orthopaedic Association | 2011 |
| 237 | Clinical Practice Guideline for Lumbar Spinal Stenosis | The Japanese Orthopaedic Association | 2011 |
| 238 | Clinical Practice Guideline for Lumbar Disc Herniation | The Japanese Orthopaedic Association | 2011 |
| 239 | Clinical Practice Guideline for Femoral Neck Fracture and Intertrochanteric Femoral Fracture | The Japanese Orthopaedic Association | 2011 |
| 240 | Evidence-based Clinical Practice Guidelines for Benign Prostatic Hyperplasia | The Japanese Urological Association | 2011 |
| 241 | Clinical Practice Guideline for　 Behcet's Disease | Research Committee on Behcet’s Disease of the Ministry of Health, Labour and Welfare of Japan | 2011 |
| 242 | Clinical Practice Guideline for Ménierè Disease | Research Committee on Vestibular Dysfunction of the Ministry of Health, Labour and Welfare of Japan | 2011 |
| 243 | Guidelines for Acute Otitis Media in Children (for patient) | Clinical Practice Guideline Development Group for the Acute Otitis Media in Children | 2011 |
| 244 | Clinical Practice Guideline for Antimicrobial Treatment of Periodontal Disease Patient | Japanese Society of Periodontology | 2011 |
| 245 | Clinical Practice Guideline for advanced trauma, Japan Advanced Trauma Evaluation and Care; JATEC | The Japanese Association for The Surgery of Trauma, Japanese Association for Acute Medicine | 2012 |
| 246 | Clinical Practice Guideline for Management of Eating Disorder | Japan Society for Eating Disorders | 2012 |
| 247 | Clinical Practice Guideline for Von Hippel-Lindau (VHL) Disease | Research Committee on Von Hippel–Lindau disease of the Ministry of Health, Labour and Welfare of Japan | 2012 |
| 248 | Guidelines for Fall Prevention in the Elderly | Research Committee on Fall Prevention in the Elderly of the Ministry of Health, Labour and Welfare of Japan | 2012 |
| 249 | Clinical Practice Guideline for Dilated Cardiomyopathy and Related Secondary Cardiomyopathy | The Japanese Circulation Society | 2012 |
| 250 | Japan Atherosclerosis Society (JAS) Guidelines for Prevention of Atherosclerotic Cardiovascular Diseases | Japan Atherosclerosis Society | 2012 |
| 251 | Clinical Practice Guideline for Diagnosis of Primary Lymphedema | Research Committee on Primary Lymphedema of the Ministry of Health, Labour and Welfare of Japan | 2012 |
| 252 | Clinical Practice Guideline for Cough | The Japanese Respiratory Society | 2012 |
| 253 | Clinical Practice Guideline for Pancreaticobiliary Maljunction | Japanese Study Group on Pancreatico biliary Maljunction, Japan Biliary Association | 2012 |
| 254 | Clinical Practice Guideline for Psychiatric Emergency Care | Japanese Association for Emergency Psychiatry | 2012 |
| 255 | Clinical Practice Guideline for Tuberculosis | The Japanese Society for Tuberculosis | 2012 |
| 256 | Japanese Pediatric Guideline for Food Allergy | Japanese Society of Pediatric Allergy and Clinical Immunology | 2012 |
| 257 | Evidence-based Guidelines for the Management of Intussusception in Children | Japanese Society of Emergency Pediatrics | 2012 |
| 258 | Clinical Practice Guideline for Cytomegalovirus Infection after Kidney Transplantation | Japanese Society for Clinical Renal Transplantation | 2012 |
| 259 | Clinical Practice Guideline for Wound and Burn | The Japanese Dermatological Association | 2012 |
| 260 | Clinical Practice Guideline for Febrile Neutropenia | Japanese Society of Medical Oncology | 2012 |
| 261 | Clinical Practice Guideline for Multiple Myeloma | Japanese Society of Myeloma | 2012 |
| 262 | Guidelines for Diagnosis and Treatment of Carcinoma of the Esophagus | The Japan Esophageal Society | 2012 |
| 263 | Evidence-based Clinical Practice Guidelines for Breast Cancer (for patient) | Japanese Breast Cancer Society | 2012 |
| 264 | JSCCR guidelines for the Clinical Practice of Hereditary Colorectal Cancer | Japanese Society for Cancer of the Colon and Rectum | 2012 |
| 265 | Clinical Practice Guideline for Prostate Cancer | The Japanese Urological Association | 2012 |
| 266 | Clinical Practice Guideline for Diagnosis of Soft-Tissue Tumor | The Japanese Orthopaedic Association | 2012 |
| 267 | Clinical Practice Guideline for Distal Radius Fracture | The Japanese Orthopaedic Association | 2012 |
| 268 | Clinical Practice Guideline for Prevention and Management of Osteoporosis | Japan Osteoporosis Society, The Japanese Society for Bone and Mineral Research, Japan Osteoporosis Foundation | 2012 |
| 269 | Clinical Practice Guideline for Low-Back Pain | The Japanese Orthopaedic Association | 2012 |
| 270 | Clinical Practice Guideline for Lateral Epicondylitis (for patient) | The Japanese Orthopaedic Association | 2012 |
| 271 | Clinical Practice Guideline for Anterior Cruciate ligament (ACL) Injuries | The Japanese Orthopaedic Association | 2012 |
| 272 | Guidelines for the Management of Atopic Dermatitis | Japanese Society of Allergology | 2012 |
| 273 | Clinical Practice Guideline for Nephrotic Syndrome | Research Committee on Refractory Nephrotic Syndrome of the Ministry of Health, Labour and Welfare of Japan | 2012 |
| 274 | Clinical Practice Guideline for Iodinated Contrast Media in Renal Impairment Patient | Japanese Society of Nephrology, Japan Radiological Society, The Japanese Circulation Society | 2012 |
| 275 | Clinical Practice Guideline for Dysuria of Spinal Cord Injury | The Japanese Continence Society, Japan Medical Society of Spinal Cord Lesion | 2012 |
| 276 | Clinical Practice Guideline for Erectile Dysfunction (ED) | The Japanese Society for Sexual Medicine | 2012 |
| 277 | Hormone Replacement Therapy Guideline | Japan Society of Obstetrics and Gynecology, Japan Society for Menopause and Women's Health | 2012 |
| 278 | Clinical Practice Guideline for Blepharospasm | The Japanese Neuro-ophthalmology Society | 2012 |
| 279 | Clinical Practice Guideline for　Dysphagia in Otorhinolaryngology Outpatient Department | The Oto-Rhino-Laryngological Society of Japan | 2012 |
| 280 | Clinical Practice Guideline for Management of Hospital Infection | Japan Infection Prevention and Control Conference for National and Public University Hospitals | 2012 |
| 281 | Clinical Practice Guideline for Parenteral and Enteral Nutrition | Japanese Society for Parenteral & Enteral Nutrition | 2013 |
| 282 | Evidence-based Practice Guideline for the Treatment of Diabetes in Japan | The Japan Diabetes Society | 2013 |
| 283 | Clinical Practice Guideline for Diagnosis and Management of Pompe Disease (glycogenosis type II) | Clinical Practice Guideline Development Group for the Pompe Disease | 2013 |
| 284 | Japanese Guidelines for Occupational Allergic Diseases | Committee for Japanese Guideline for Diagnosis and Management of Occupational Allergic Disease | 2013 |
| 285 | Clinical Practice Guideline for Safety of Latex Allergy | Japanese Society of Latex Allergy | 2013 |
| 286 | Clinical Practice Guideline for Pharmacotherapy of Heart Disease in Children | The Japanese Circulation Society | 2013 |
| 287 | Clinical Practice Guideline for Prevention and Management of Asthma | Research Committee on Immunity and Allergy of the Ministry of Health, Labour and Welfare of Japan | 2013 |
| 288 | Clinical Practice Guideline for Diagnosis and Treatment of Chronic Obstructive Pulmonary Disease (COPD) | The Japanese Respiratory Society | 2013 |
| 289 | Clinical Practice Guideline for Acute Cholangitis and Acute Cholecystitis | Japanese Society for Abdominal Emergency Medicine, Japanese Society of Hepato-Biliary-Pancreatic Surgery, Japan Biliary Association, Japan Society for Surgical Infection, Japan Radiological Society | 2013 |
| 290 | Clinical Practice Guideline for Fibromyalgia | Japan College of Fibromyalgia Investigation | 2013 |
| 291 | Clinical Practice Guideline for　Chronic Headache | Japanese Society of Neurology, The Japanese Headache Society | 2013 |
| 292 | Practical Guideline for Guillain-Barré Syndrome and Fisher Syndrome | Japanese Society of Neurology | 2013 |
| 293 | Clinical Practice Guideline for Chronic Inflammatory Demyelinating Polyradiculoneuropathy and Multifocal Motor Neuropathy | Japanese Society of Neurology | 2013 |
| 294 | Clinical Practice Guideline for Amyotrophic Lateral Sclerosis | Japanese Society of Neurology | 2013 |
| 295 | Clinical Practice Guideline for Progressive Multifocal Leukoencephalopathy (PML) | Research Committee on Prion Disease and Slow Virus Infection of the Ministry of Health, Labour and Welfare of Japan | 2013 |
| 296 | Clinical Practice Guideline for Major Depressive Disorder and Bipolar Disorder | Japanese Society of Mood Disorders | 2013 |
| 297 | Clinical Practice Guideline for Diagnosis and Management of Sexually Transmitted Disease | Japanese Society for Sexually Transmitted Infections | 2013 |
| 298 | Clinical Practice Guideline for Management of MRSA Infection | Japanese Society of Chemotherapy, The Japanese Association for Infectious Diseases | 2013 |
| 299 | Clinical Practice Guideline for Sepsis | The Japanese Society of Intensive Care Medicine | 2013 |
| 300 | Clinical Practice Guideline for Asthma (for patient) | Japanese Society of Pediatric Allergy and Clinical Immunology | 2013 |
| 301 | Clinical Practice Guideline for Chronic Functional Constipation in Children | The Japanese Society for Pediatric Gastroenterology, Hepatology and Nutrition, Japanese Society for Pediatric Neurogastroenterology | 2013 |
| 302 | Clinical Practice Guideline for Infantile Idiopathic Nephrotic Syndrome | The Japanese Society for Pediatric Nephrology | 2013 |
| 303 | Clinical Practice Guideline for Management of Pain Clinic | Japan Society of Pain Clinicians | 2013 |
| 304 | Clinical Practice Guideline for Fluid Therapy of Terminal Cancer Patient | Japanese Society for Palliative Medicine | 2013 |
| 305 | Clinical Practice Guideline for Rehabilitation of Cancer | Japanese Association of Rehabilitation Medicine | 2013 |
| 306 | Clinical Practice Guideline for Tumour Lysis Syndrome (TLS) | Japanese Society of Medical Oncology | 2013 |
| 307 | Clinical Practice Guideline for Proper Use of Granular Colony Stimulating Factor (G-CSF) | Japan Society of Clinical Oncology | 2013 |
| 308 | Evidence-based Clinical Practice Guidelines for Breast Cancer, Treatment | Japanese Breast Cancer Society | 2013 |
| 309 | Evidence-based Clinical Practice Guidelines for Breast Cancer, Epidemiology and Diagnosis | Japanese Breast Cancer Society | 2013 |
| 310 | Evidence-based Clinical Practice Guidelines for Pancreatic Cancer | Japan Pancreas Society | 2013 |
| 311 | Clinical Practice Guideline for Thyroid Nodule | Japan Thyroid Association | 2013 |
| 312 | Clinical Practice Guideline for Multiple Endocrine Neoplasia | Clinical Practice Guideline Development Group for the Multiple Endocrine Neoplasia | 2013 |
| 313 | Clinical Practice Guideline for　Uterine Cancer | The Japan Society of Gynecologic Oncology | 2013 |
| 314 | Clinical Practice Guideline for Head and Neck Cancer | Japan Society for Head and Neck Cancer | 2013 |
| 315 | Evidence-based Clinical Practice Guidelines for Oral Cancer | Japanese Society of Oral Oncology, Japanese Society of Oral and Maxillofacial Surgeons | 2013 |
| 316 | Clinical Practice Guideline for Treatment and Management of Severe Head Injury | Clinical Practice Guideline Development Group for the Treatment and Management of Severe Head Injury | 2013 |
| 317 | Clinical Practice Guideline for Management of Stereotactic and Functional Neurosurgery | Japan Society for Stereotactic and Functional Neurosurgery | 2013 |
| 318 | Clinical Practice Guideline for Awake Surgery | Japanese Society of Awake Surgery | 2013 |
| 319 | Clinical Practice Guideline for Urolithiasis | The Japanese Urological Association, Japanese Society of Endourology and ESWL, Japanese Society on Urolithiasis Research | 2013 |
| 320 | Evidence-based Clinical Practice Guideline for CKD | Japanese Society of Nephrology | 2013 |
| 321 | Clinical Practice Guideline for Gynecological Endoscopic Surgery | The Japan Society of Gynecologic and Obstetric Endoscopy and Minimally Invasive Therapy | 2013 |
| 322 | Clinical Practice Guideline for Lower Urinary Tract Symptom in Woman | The Japanese Continence Society | 2013 |
| 323 | Clinical Practice Guideline for Pregnancy and Delivery | Research Committee on Pregnancy and Childbirth of the Ministry of Health, Labour and Welfare of Japan | 2013 |
| 324 | Guidelines for Acute Otitis Media in Children | Japan Otological Society, Japan Society for Pediatric ORL, Japan Society for Infectious Diseases in Otolaryngology | 2013 |
| 325 | Allergic Rhinitis Guide | Clinical Practice Guideline Development Group for the Management of Allergic Rhinitis | 2013 |
| 326 | Practical Guideline for the Management of Allergic Rhinitis in Japan <PG-MARJ> | Clinical Practice Guideline Development Group for the Management of Allergic Rhinitis | 2013 |
| 327 | Clinical Practice Guideline for Regenerative Treatment of Periodontal Disease Patient | Japanese Society of Periodontology | 2013 |
| 328 | Clinical Practice Guideline for Tooth Extraction of Antithrombotic Therapy Patient | Japanese Society of Dentistry for Medically Compromised Patient, Japanese Society of Oral and Maxillofacial Surgeons, Japanese Society of Gerodontology | 2013 |
| 329 | Clinical Practice Guideline for Implant Treatment of Periodontal Disease Patient | The Japanese Academy of Clinical Periodontology | 2013 |
| 330 | Clinical Practice Guideline for Neuro-Behcet Disease | Research Committee on Behcet’s Disease of the Ministry of Health, Labour and Welfare of Japan | 2014 |
| 331 | Anaphylaxis Guideline | Japanese Society of Allergology | 2014 |
| 332 | Clinical Practice Guideline for ANCA-associated Vasculitis | Research Committee on Intractable Vasculitis and Progressive Renal Disorder of the Ministry of Health, Labour and Welfare of Japan | 2014 |
| 333 | Japanese Society of Hypertension Guidelines for the Management of Hypertension (JSH 2014) | The Japanese Society of Hypertension | 2014 |
| 334 | Clinical Practice Guideline for Management of Respiratory Infection | The Japanese Association for Infectious Diseases, Japanese Society of Chemotherapy | 2014 |
| 335 | Clinical Practice Guideline for Mycoplasma Pneumonia | The Japanese Society of Mycoplasmology | 2014 |
| 336 | Evidence-based Clinical Practice Guidelines for Functional Dyspepsia | The Japanese Society of Gastroenterology | 2014 |
| 337 | Evidence-based Clinical Practice Guidelines for Irritable Bowel Syndrome | The Japanese Society of Gastroenterology | 2014 |
| 338 | Clinical Practice Guideline for Autoimmune Hepatitis （AIH) | Research Committee on Intractable Hepatic and Biliary Tract Disease of the Ministry of Health, Labour and Welfare of Japan | 2014 |
| 339 | Clinical Practice Guideline for Non-Alcoholic Fatty Liver Disease (NAFLD) and Non-Alcoholic Steatohepatitis (NASH) | The Japanese Society of Gastroenterology | 2014 |
| 340 | Clinical Practice Guideline for Rheumatoid Arthritis | Japan College of Rheumatology | 2014 |
| 341 | Clinical Practice Guideline for　Chronic Headache (for patient) | The Japanese Headache Society | 2014 |
| 342 | Practical Guideline for Duchenne Muscular Dystrophy(DMD) | Japanese Society of Neurology, The Japanese Society of Child Neurology, National Center of Neurology and Psychiatry | 2014 |
| 343 | Clinical Practice Guideline for Myasthenia Gravis | Japanese Society of Neurology | 2014 |
| 344 | Clinical Practice Guideline for Pulmonary Rehabilitation of Neuromuscular Disease and Spinal Cord Injury | Japanese Association of Rehabilitation Medicine | 2014 |
| 345 | Clinical Practice Guideline for Prion Disease | Research Committee on Prion Disease and Slow Virus Infection of the Ministry of Health, Labour and Welfare of Japan | 2014 |
| 346 | Clinical Practice Guideline for Proper Use of Hypnotics | Research Committee on Proper Use of Sleeping Pills of the Ministry of Health, Labour and Welfare of Japan , The Japanese Society of Sleep Research | 2014 |
| 347 | Guidelines for Management of Deep-Seated Mycoses | The Japanese Society for Medical Mycology | 2014 |
| 348 | Clinical Practice Guideline for Management of MRSA Infection | Japanese Society of Chemotherapy, The Japanese Association for Infectious Diseases | 2014 |
| 349 | Clinical Practice Guideline for Cough in Children | Japanese Society of Pediatric Pulmonology | 2014 |
| 350 | Guidelines for the Interventional Pain Treatment | Japan Society of Pain Clinicians | 2014 |
| 351 | Clinical Practice Guideline for Therapeutic Drug Monitoring (TDM) of Immunosuppressant | The Japanese Society of Therapeutic Drug Monitoring, The Japan Society for Transplantation | 2014 |
| 352 | Clinical Practice Guideline for Expert Trauma, Japan Expert Trauma Evaluation and Care；JETEC | The Japanese Association for The Surgery of Trauma | 2014 |
| 353 | Guidelines for the Interventional treatment of Cancer Pain | Japan Society of Pain Clinicians | 2014 |
| 354 | Clinical Guidelines for Cancer Pain Management | Japanese Society for Palliative Medicine | 2014 |
| 355 | Clinical Practice Guideline for Pregnancy and Delivery and Reproductive Medicine of Breast Cancer Patient | Research Committee on Support System for Fertility Preservation of Breast Cancer Patient of the Ministry of Health, Labour and Welfare of Japan, Japan Society for Fertility Preservation | 2014 |
| 356 | Evidence-based Clinical Practice Guidelines for Breast Cancer (for patient) | Japanese Breast Cancer Society | 2014 |
| 357 | Evidence-based Clinical Practice Guidelines for Lung Cancer | The Japan Lung Cancer Society | 2014 |
| 358 | Clinical Practice Guideline for GIST | Japan Society of Clinical Oncology, Japanese Gastric Cancer Association, Japanese Study Group on GIST | 2014 |
| 359 | Japanese Gastric Cancer Treatment Guidelines 2014 (ver. 4) | Japanese Gastric Cancer Association | 2014 |
| 360 | JSCCR guidelines for the Treatment of Colorectal Cancer | Japanese Society for Cancer of the Colon and Rectum | 2014 |
| 361 | JSCCR guidelines for the Treatment of Colorectal Cancer (for patient) | Japanese Society for Cancer of the Colon and Rectum | 2014 |
| 362 | Clinical Practice Guideline for　 Colon Polyp | The Japanese Society of Gastroenterology | 2014 |
| 363 | Evidence-based Clinical Practice Guidelines for Biliary Tract Cancer | Japanese Society of Hepato-Biliary-Pancreatic Surgery | 2014 |
| 364 | Clinical Practice Guideline for Renal Pelvic Cancer and Ureteral Cancer | The Japanese Urological Association | 2014 |
| 365 | Clinical Practice Guideline for Anal Disease | The Japan Society of Coloproctology | 2014 |
| 366 | Clinical Practice Guideline for Hallux Valgus | The Japanese Orthopaedic Association | 2014 |
| 367 | Clinical Practice Guideline for Rehabilitation of Cerebral Palsy | Japanese Association of Rehabilitation Medicine | 2014 |
| 368 | Clinical Practice Guideline for Polycystic Kidney Disease (PKD) | Research Committee on Progressive Renal Disorder of the Ministry of Health, Labour and Welfare of Japan | 2014 |
| 369 | Clinical Practice Guideline for Diagnosis and Management of Hemolytic Uremic Syndrome | Clinical Practice Guideline Development Group for the Diagnosis and Management of Hemolytic-Uremic Syndrome | 2014 |
| 370 | Clinical Practice Guideline for Acute Scrotum | The Japanese Urological Association | 2014 |
| 371 | Guideline for Gynecological Practice in Japan 2014 | Japan Society of Obstetrics and Gynecology, Japan Association of Obstetricians and Gynecologists | 2014 |
| 372 | Guideline for Obstetrical Practice in Japan 2014 | Japan Society of Obstetrics and Gynecology, Japan Association of Obstetricians and Gynecologists | 2014 |
| 373 | Clinical Practice Guideline for Regenerative Treatment of Periodontal Disease Patient | Japanese Society of Periodontology | 2014 |
