# Supplementary material for: Quality assessment of clinical practice guidelines using the AGREE instrument in Japan: A time trend analysis
Source: PLoS One. 2019 May 2;14(5):e0216346. doi: 10.1371/journal.pone.0216346 (PMC6497296; doi:10.1371/journal.pone.0216346)
Supplement: S2 Table — (DOCX) [file pone.0216346.s002.docx]

Table S2. Clinical practice guidelines’ standardized scores on each domain of AGREE (%).

| No | #1. Scope and Purpose | #2. Stakeholder involvement | #3. Rigour of Development | #4. Clarity of Presentation | #5. Applicability | #6. Editorial Independence | Total |
| --- | --- | --- | --- | --- | --- | --- | --- |
| 1 | 88.9 | 47.2 | 28.6 | 58.3 | 11.1 | 33.3 | 43.0 |
| 2 | 100.0 | 36.1 | 39.7 | 75.0 | 33.3 | 33.3 | 51.7 |
| 3 | 100.0 | 30.6 | 28.6 | 52.8 | 14.8 | 16.7 | 39.6 |
| 4 | 85.2 | 33.3 | 22.2 | 63.9 | 63.0 | 33.3 | 45.9 |
| 5 | 85.2 | 50.0 | 52.4 | 66.7 | 11.1 | 44.4 | 52.7 |
| 6 | 63.0 | 41.7 | 15.9 | 41.7 | 37.0 | 5.6 | 32.9 |
| 7 | 100.0 | 33.3 | 41.3 | 61.1 | 37.0 | 50.0 | 51.2 |
| 8 | 100.0 | 50.0 | 65.1 | 88.9 | 59.3 | 50.0 | 69.1 |
| 9 | 81.5 | 63.9 | 65.1 | 91.7 | 11.1 | 38.9 | 62.3 |
| 10 | 51.9 | 30.6 | 33.3 | 61.1 | 22.2 | 5.6 | 36.2 |
| 11 | 74.1 | 52.8 | 25.4 | 55.6 | 29.6 | 11.1 | 41.1 |
| 12 | 66.7 | 50.0 | 50.8 | 72.2 | 37.0 | 22.2 | 52.2 |
| 13 | 92.6 | 52.8 | 31.7 | 33.3 | 25.9 | 44.4 | 44.0 |
| 14 | 96.3 | 58.3 | 23.8 | 77.8 | 11.1 | 50.0 | 49.3 |
| 15 | 77.8 | 47.2 | 68.3 | 61.1 | 33.3 | 22.2 | 56.0 |
| 16 | 88.9 | 50.0 | 25.4 | 50.0 | 11.1 | 33.3 | 41.1 |
| 17 | 88.9 | 44.4 | 14.3 | 50.0 | 7.4 | 50.0 | 37.7 |
| 18 | 96.3 | 47.2 | 22.2 | 77.8 | 7.4 | 44.4 | 45.9 |
| 19 | 66.7 | 52.8 | 12.7 | 52.8 | 29.6 | 16.7 | 36.2 |
| 20 | 66.7 | 33.3 | 6.3 | 55.6 | 44.4 | 16.7 | 33.3 |
| 21 | 70.4 | 69.4 | 66.7 | 66.7 | 37.0 | 22.2 | 59.9 |
| 22 | 55.6 | 36.1 | 33.3 | 55.6 | 29.6 | 0.0 | 37.2 |
| 23 | 74.1 | 33.3 | 42.9 | 77.8 | 33.3 | 0.0 | 46.4 |
| 24 | 88.9 | 77.8 | 93.7 | 97.2 | 44.4 | 44.4 | 80.2 |
| 25 | 77.8 | 44.4 | 15.9 | 55.6 | 3.7 | 50.0 | 37.2 |
| 26 | 96.3 | 41.7 | 73.0 | 97.2 | 44.4 | 50.0 | 69.1 |
| 27 | 55.6 | 61.1 | 25.4 | 41.7 | 37.0 | 5.6 | 38.2 |
| 28 | 63.0 | 55.6 | 68.3 | 66.7 | 29.6 | 0.0 | 54.1 |
| 29 | 59.3 | 47.2 | 31.7 | 44.4 | 29.6 | 16.7 | 38.6 |
| 30 | 77.8 | 44.4 | 34.9 | 55.6 | 25.9 | 0.0 | 41.5 |
| 31 | 55.6 | 38.9 | 15.9 | 36.1 | 40.7 | 16.7 | 31.9 |
| 32 | 66.7 | 50.0 | 77.8 | 80.6 | 14.8 | 5.6 | 57.5 |
| 33 | 96.3 | 55.6 | 54.0 | 55.6 | 33.3 | 50.0 | 57.0 |
| 34 | 88.9 | 36.1 | 17.5 | 72.2 | 11.1 | 33.3 | 40.1 |
| 35 | 100.0 | 38.9 | 76.2 | 80.6 | 25.9 | 33.3 | 63.3 |
| 36 | 100.0 | 44.4 | 33.3 | 77.8 | 40.7 | 33.3 | 52.7 |
| 37 | 92.6 | 55.6 | 34.9 | 72.2 | 25.9 | 33.3 | 51.2 |
| 38 | 96.3 | 44.4 | 46.0 | 75.0 | 55.6 | 50.0 | 58.9 |
| 39 | 74.1 | 36.1 | 14.3 | 16.7 | 11.1 | 50.0 | 29.0 |
| 40 | 100.0 | 66.7 | 63.5 | 88.9 | 0.0 | 22.2 | 61.4 |
| 41 | 96.3 | 61.1 | 93.7 | 80.6 | 40.7 | 38.9 | 74.4 |
| 42 | 81.5 | 52.8 | 6.3 | 77.8 | 0.0 | 27.8 | 37.7 |
| 43 | 100.0 | 52.8 | 23.8 | 77.8 | 7.4 | 44.4 | 47.8 |
| 44 | 40.7 | 58.3 | 68.3 | 44.4 | 55.6 | 11.1 | 52.2 |
| 45 | 59.3 | 52.8 | 74.6 | 75.0 | 37.0 | 61.1 | 62.8 |
| 46 | 66.7 | 52.8 | 60.3 | 88.9 | 44.4 | 11.1 | 58.5 |
| 47 | 70.4 | 25.0 | 55.6 | 44.4 | 25.9 | 0.0 | 41.5 |
| 48 | 92.6 | 47.2 | 73.0 | 72.2 | 25.9 | 0.0 | 58.5 |
| 49 | 51.9 | 36.1 | 47.6 | 50.0 | 18.5 | 0.0 | 38.6 |
| 50 | 51.9 | 66.7 | 38.1 | 58.3 | 14.8 | 44.4 | 45.9 |
| 51 | 66.7 | 69.4 | 30.2 | 63.9 | 29.6 | 11.1 | 45.9 |
| 52 | 77.8 | 44.4 | 69.8 | 63.9 | 11.1 | 16.7 | 53.1 |
| 53 | 74.1 | 25.0 | 73.0 | 75.0 | 33.3 | 11.1 | 54.6 |
| 54 | 74.1 | 66.7 | 76.2 | 77.8 | 51.9 | 11.1 | 65.7 |
| 55 | 66.7 | 44.4 | 20.6 | 50.0 | 22.2 | 11.1 | 35.3 |
| 56 | 66.7 | 63.9 | 14.3 | 41.7 | 14.8 | 27.8 | 35.7 |
| 57 | 77.8 | 47.2 | 54.0 | 41.7 | 25.9 | 22.2 | 47.3 |
| 58 | 96.3 | 50.0 | 93.7 | 69.4 | 40.7 | 50.0 | 71.5 |
| 59 | 70.4 | 41.7 | 11.1 | 36.1 | 14.8 | 16.7 | 29.5 |
| 60 | 66.7 | 52.8 | 15.9 | 50.0 | 37.0 | 22.2 | 38.2 |
| 61 | 66.7 | 41.7 | 34.9 | 52.8 | 11.1 | 22.2 | 39.1 |
| 62 | 96.3 | 55.6 | 96.8 | 63.9 | 22.2 | 50.0 | 70.0 |
| 63 | 85.2 | 47.2 | 93.7 | 69.4 | 29.6 | 33.3 | 66.7 |
| 64 | 88.9 | 61.1 | 84.1 | 72.2 | 25.9 | 27.8 | 66.2 |
| 65 | 85.2 | 61.1 | 87.3 | 72.2 | 22.2 | 27.8 | 66.2 |
| 66 | 77.8 | 55.6 | 87.3 | 75.0 | 29.6 | 27.8 | 65.7 |
| 67 | 74.1 | 55.6 | 90.5 | 72.2 | 22.2 | 27.8 | 64.7 |
| 68 | 85.2 | 72.2 | 74.6 | 75.0 | 18.5 | 22.2 | 63.8 |
| 69 | 85.2 | 47.2 | 69.8 | 75.0 | 18.5 | 33.3 | 58.9 |
| 70 | 88.9 | 41.7 | 81.0 | 72.2 | 22.2 | 27.8 | 61.4 |
| 71 | 92.6 | 41.7 | 87.3 | 72.2 | 22.2 | 22.2 | 63.3 |
| 72 | 74.1 | 50.0 | 68.3 | 55.6 | 29.6 | 22.2 | 54.6 |
| 73 | 85.2 | 44.4 | 74.6 | 86.1 | 29.6 | 44.4 | 64.3 |
| 74 | 96.3 | 72.2 | 98.4 | 86.1 | 48.1 | 100.0 | 85.0 |
| 75 | 77.8 | 50.0 | 55.6 | 69.4 | 14.8 | 22.2 | 51.7 |
| 76 | 92.6 | 55.6 | 73.0 | 88.9 | 11.1 | 27.8 | 63.3 |
| 77 | 85.2 | 44.4 | 31.7 | 44.4 | 14.8 | 27.8 | 40.6 |
| 78 | 70.4 | 47.2 | 38.1 | 55.6 | 11.1 | 22.2 | 42.0 |
| 79 | 88.9 | 69.4 | 17.5 | 66.7 | 14.8 | 27.8 | 44.9 |
| 80 | 88.9 | 58.3 | 47.6 | 66.7 | 22.2 | 11.1 | 51.7 |
| 81 | 96.3 | 61.1 | 12.7 | 66.7 | 18.5 | 27.8 | 43.5 |
| 82 | 88.9 | 61.1 | 57.1 | 58.3 | 7.4 | 27.8 | 53.1 |
| 83 | 96.3 | 50.0 | 71.4 | 72.2 | 14.8 | 27.8 | 59.9 |
| 84 | 77.8 | 50.0 | 69.8 | 72.2 | 29.6 | 22.2 | 58.5 |
| 85 | 100.0 | 66.7 | 65.1 | 83.3 | 29.6 | 22.2 | 64.7 |
| 86 | 92.6 | 61.1 | 93.7 | 83.3 | 29.6 | 44.4 | 73.4 |
| 87 | 81.5 | 41.7 | 52.4 | 61.1 | 18.5 | 22.2 | 48.8 |
| 88 | 88.9 | 47.2 | 38.1 | 50.0 | 11.1 | 22.2 | 43.5 |
| 89 | 85.2 | 63.9 | 47.6 | 63.9 | 18.5 | 38.9 | 53.6 |
| 90 | 96.3 | 47.2 | 71.4 | 80.6 | 18.5 | 27.8 | 61.4 |
| 91 | 85.2 | 58.3 | 84.1 | 91.7 | 25.9 | 50.0 | 70.5 |
| 92 | 100.0 | 72.2 | 93.7 | 83.3 | 33.3 | 44.4 | 76.8 |
| 93 | 100.0 | 63.9 | 90.5 | 97.2 | 14.8 | 27.8 | 72.9 |
| 94 | 88.9 | 58.3 | 68.3 | 80.6 | 29.6 | 22.2 | 62.3 |
| 95 | 74.1 | 63.9 | 22.2 | 36.1 | 18.5 | 27.8 | 38.6 |
| 96 | 92.6 | 61.1 | 30.2 | 58.3 | 14.8 | 27.8 | 46.4 |
| 97 | 100.0 | 83.3 | 60.3 | 97.2 | 25.9 | 27.8 | 68.6 |
| 98 | 81.5 | 72.2 | 12.7 | 80.6 | 25.9 | 27.8 | 46.9 |
| 99 | 74.1 | 55.6 | 12.7 | 69.4 | 44.4 | 27.8 | 43.5 |
| 100 | 66.7 | 52.8 | 27.0 | 55.6 | 14.8 | 27.8 | 40.1 |
| 101 | 85.2 | 61.1 | 22.2 | 38.9 | 25.9 | 22.2 | 40.6 |
| 102 | 100.0 | 52.8 | 88.9 | 77.8 | 59.3 | 100.0 | 79.2 |
| 103 | 85.2 | 72.2 | 96.8 | 97.2 | 63.0 | 22.2 | 80.2 |
| 104 | 96.3 | 41.7 | 71.4 | 75.0 | 37.0 | 22.2 | 61.4 |
| 105 | 96.3 | 91.7 | 92.1 | 88.9 | 48.1 | 66.7 | 84.1 |
| 106 | 88.9 | 72.2 | 71.4 | 75.0 | 22.2 | 22.2 | 63.8 |
| 107 | 96.3 | 72.2 | 88.9 | 88.9 | 18.5 | 22.2 | 72.0 |
| 108 | 100.0 | 38.9 | 84.1 | 75.0 | 25.9 | 27.8 | 64.3 |
| 109 | 85.2 | 41.7 | 30.2 | 47.2 | 40.7 | 22.2 | 43.0 |
| 110 | 88.9 | 41.7 | 69.8 | 80.6 | 29.6 | 22.2 | 59.9 |
| 111 | 70.4 | 36.1 | 46.0 | 66.7 | 18.5 | 38.9 | 46.9 |
| 112 | 92.6 | 52.8 | 65.1 | 86.1 | 33.3 | 22.2 | 62.3 |
| 113 | 100.0 | 50.0 | 90.5 | 91.7 | 33.3 | 22.2 | 71.5 |
| 114 | 81.5 | 41.7 | 15.9 | 25.0 | 29.6 | 22.2 | 32.9 |
| 115 | 88.9 | 69.4 | 25.4 | 33.3 | 25.9 | 22.2 | 42.5 |
| 116 | 88.9 | 44.4 | 68.3 | 72.2 | 14.8 | 22.2 | 56.5 |
| 117 | 11.1 | 77.8 | 69.8 | 77.8 | 29.6 | 22.2 | 66.7 |
| 118 | 92.6 | 50.0 | 90.5 | 88.9 | 25.9 | 100.0 | 75.8 |
| 119 | 88.9 | 72.2 | 34.9 | 58.3 | 29.6 | 22.2 | 50.7 |
| 120 | 96.3 | 41.7 | 87.3 | 66.7 | 29.6 | 22.2 | 63.8 |
| 121 | 88.9 | 55.6 | 50.8 | 83.3 | 18.5 | 22.2 | 55.6 |
| 122 | 77.8 | 44.4 | 22.2 | 22.2 | 22.2 | 22.2 | 33.3 |
| 123 | 44.4 | 36.1 | 11.1 | 33.3 | 25.9 | 22.2 | 26.6 |
| 124 | 88.9 | 41.7 | 85.7 | 72.2 | 25.9 | 22.2 | 62.8 |
| 125 | 100.0 | 75.0 | 85.7 | 88.9 | 37.0 | 22.2 | 74.4 |
| 126 | 85.2 | 50.0 | 77.8 | 58.3 | 11.1 | 100.0 | 63.8 |
| 127 | 100.0 | 66.7 | 93.7 | 86.1 | 40.7 | 100.0 | 82.1 |
| 128 | 96.3 | 50.0 | 69.8 | 80.6 | 14.8 | 38.9 | 61.8 |
| 129 | 100.0 | 55.6 | 98.4 | 83.3 | 59.3 | 55.6 | 79.7 |
| 130 | 100.0 | 55.6 | 87.3 | 83.3 | 33.3 | 94.4 | 76.3 |
| 131 | 96.3 | 80.6 | 7.9 | 75.0 | 22.2 | 38.9 | 48.3 |
| 132 | 100.0 | 55.6 | 66.7 | 88.9 | 22.2 | 38.9 | 64.7 |
| 133 | 100.0 | 58.3 | 79.4 | 63.9 | 55.6 | 22.2 | 67.6 |
| 134 | 100.0 | 72.2 | 92.1 | 86.1 | 22.2 | 38.9 | 74.9 |
| 135 | 96.3 | 75.0 | 95.2 | 83.3 | 22.2 | 38.9 | 75.4 |
| 136 | 100.0 | 75.0 | 88.9 | 86.1 | 18.5 | 33.3 | 73.4 |
| 137 | 100.0 | 86.1 | 92.1 | 88.9 | 18.5 | 38.9 | 77.3 |
| 138 | 96.3 | 72.2 | 55.6 | 55.6 | 51.9 | 77.8 | 65.2 |
| 139 | 100.0 | 83.3 | 55.6 | 63.9 | 55.6 | 38.9 | 66.2 |
| 140 | 81.5 | 55.6 | 9.5 | 44.4 | 22.2 | 38.9 | 37.2 |
| 141 | 92.6 | 58.3 | 23.8 | 61.1 | 33.3 | 33.3 | 47.3 |
| 142 | 100.0 | 83.3 | 33.3 | 83.3 | 33.3 | 50.0 | 60.9 |
| 143 | 100.0 | 86.1 | 50.8 | 88.9 | 51.9 | 38.9 | 69.1 |
| 144 | 100.0 | 83.3 | 52.4 | 63.9 | 33.3 | 22.2 | 60.9 |
| 145 | 96.3 | 77.8 | 54.0 | 80.6 | 59.3 | 44.4 | 68.1 |
| 146 | 100.0 | 52.8 | 88.9 | 77.8 | 59.3 | 100.0 | 79.2 |
| 147 | 88.9 | 86.1 | 55.6 | 80.6 | 44.4 | 94.4 | 71.5 |
| 148 | 96.3 | 55.6 | 90.5 | 72.2 | 14.8 | 33.3 | 67.1 |
| 149 | 77.8 | 69.4 | 36.5 | 69.4 | 29.6 | 33.3 | 52.2 |
| 150 | 81.5 | 72.2 | 42.9 | 63.9 | 48.1 | 27.8 | 56.0 |
| 151 | 96.3 | 50.0 | 95.2 | 66.7 | 55.6 | 94.4 | 77.3 |
| 152 | 81.5 | 50.0 | 50.8 | 58.3 | 44.4 | 33.3 | 53.6 |
| 153 | 96.3 | 52.8 | 88.9 | 66.7 | 40.7 | 94.4 | 73.9 |
| 154 | 96.3 | 83.3 | 93.7 | 94.4 | 59.3 | 100.0 | 88.4 |
| 155 | 96.3 | 47.2 | 92.1 | 69.4 | 55.6 | 94.4 | 76.3 |
| 156 | 100.0 | 41.7 | 95.2 | 69.4 | 51.9 | 94.4 | 76.3 |
| 157 | 85.2 | 47.2 | 11.1 | 41.7 | 66.7 | 33.3 | 41.5 |
| 158 | 92.6 | 36.1 | 60.3 | 69.4 | 29.6 | 33.3 | 55.6 |
| 159 | 100.0 | 69.4 | 90.5 | 83.3 | 40.7 | 100.0 | 81.2 |
| 160 | 100.0 | 66.7 | 23.8 | 50.0 | 3.7 | 33.3 | 44.0 |
| 161 | 100.0 | 66.7 | 93.7 | 91.7 | 14.8 | 50.0 | 75.4 |
| 162 | 100.0 | 75.0 | 92.1 | 88.9 | 40.7 | 100.0 | 83.6 |
| 163 | 85.2 | 44.4 | 71.4 | 63.9 | 37.0 | 44.4 | 60.4 |
| 164 | 100.0 | 55.6 | 87.3 | 91.7 | 40.7 | 44.4 | 74.4 |
| 165 | 100.0 | 69.4 | 84.1 | 86.1 | 55.6 | 38.9 | 76.3 |
| 166 | 100.0 | 41.7 | 55.6 | 69.4 | 25.9 | 38.9 | 56.0 |
| 167 | 100.0 | 66.7 | 95.2 | 80.6 | 44.4 | 44.4 | 77.3 |
| 168 | 92.6 | 47.2 | 61.9 | 69.4 | 33.3 | 44.4 | 59.4 |
| 169 | 88.9 | 69.4 | 15.9 | 47.2 | 29.6 | 44.4 | 44.4 |
| 170 | 100.0 | 66.7 | 95.2 | 88.9 | 59.3 | 94.4 | 85.0 |
| 171 | 96.3 | 55.6 | 85.7 | 86.1 | 40.7 | 94.4 | 76.8 |
| 172 | 96.3 | 52.8 | 60.3 | 61.1 | 22.2 | 22.2 | 55.6 |
| 173 | 92.6 | 44.4 | 47.6 | 44.4 | 33.3 | 38.9 | 49.8 |
| 174 | 100.0 | 52.8 | 68.3 | 77.8 | 22.2 | 38.9 | 62.8 |
| 175 | 100.0 | 77.8 | 96.8 | 91.7 | 51.9 | 100.0 | 87.4 |
| 176 | 96.3 | 50.0 | 88.9 | 77.8 | 37.0 | 50.0 | 71.0 |
| 177 | 100.0 | 88.9 | 50.8 | 80.6 | 37.0 | 44.4 | 66.7 |
| 178 | 70.4 | 72.2 | 17.5 | 58.3 | 14.8 | 38.9 | 42.5 |
| 179 | 100.0 | 69.4 | 90.5 | 86.1 | 40.7 | 83.3 | 80.2 |
| 180 | 100.0 | 61.1 | 28.6 | 69.4 | 22.2 | 38.9 | 50.7 |
| 181 | 92.6 | 38.9 | 38.1 | 69.4 | 37.0 | 94.4 | 55.6 |
| 182 | 92.6 | 77.8 | 79.4 | 88.9 | 48.1 | 88.9 | 79.2 |
| 183 | 100.0 | 86.1 | 96.8 | 91.7 | 40.7 | 94.4 | 87.0 |
| 184 | 85.2 | 36.1 | 30.2 | 50.0 | 40.7 | 100.0 | 49.3 |
| 185 | 96.3 | 66.7 | 27.0 | 38.9 | 22.2 | 44.4 | 45.9 |
| 186 | 100.0 | 66.7 | 17.5 | 69.4 | 3.7 | 44.4 | 46.4 |
| 187 | 100.0 | 69.4 | 14.3 | 72.2 | 3.7 | 44.4 | 46.4 |
| 188 | 85.2 | 50.0 | 76.2 | 80.6 | 48.1 | 100.0 | 72.0 |
| 189 | 92.6 | 33.3 | 7.9 | 50.0 | 18.5 | 100.0 | 40.1 |
| 190 | 100.0 | 63.9 | 11.1 | 83.3 | 22.2 | 44.4 | 48.8 |
| 191 | 96.3 | 47.2 | 85.7 | 75.0 | 55.6 | 94.4 | 75.4 |
| 192 | 100.0 | 61.1 | 17.5 | 80.6 | 18.5 | 33.3 | 48.3 |
| 193 | 88.9 | 50.0 | 54.0 | 72.2 | 66.7 | 44.4 | 61.8 |
| 194 | 100.0 | 69.4 | 88.9 | 75.0 | 33.3 | 94.4 | 77.8 |
| 195 | 100.0 | 72.2 | 79.4 | 75.0 | 37.0 | 61.1 | 72.9 |
| 196 | 100.0 | 72.2 | 90.5 | 91.7 | 33.3 | 94.4 | 81.6 |
| 197 | 88.9 | 55.6 | 33.3 | 33.3 | 29.6 | 22.2 | 43.0 |
| 198 | 92.6 | 55.6 | 60.3 | 47.2 | 18.5 | 44.4 | 54.6 |
| 199 | 100.0 | 69.4 | 88.9 | 91.7 | 29.6 | 100.0 | 80.7 |
| 200 | 96.3 | 86.1 | 81.0 | 61.1 | 18.5 | 100.0 | 73.9 |
| 201 | 100.0 | 41.7 | 71.4 | 83.3 | 29.6 | 61.1 | 65.7 |
| 202 | 100.0 | 41.7 | 84.1 | 88.9 | 40.7 | 61.1 | 72.0 |
| 203 | 100.0 | 69.4 | 92.1 | 83.3 | 44.4 | 44.4 | 77.3 |
| 204 | 100.0 | 63.9 | 74.6 | 80.6 | 33.3 | 50.0 | 69.6 |
| 205 | 96.3 | 72.2 | 41.3 | 83.3 | 29.6 | 44.4 | 59.9 |
| 206 | 100.0 | 66.7 | 93.7 | 83.3 | 37.0 | 83.3 | 79.7 |
| 207 | 100.0 | 55.6 | 85.7 | 86.1 | 40.7 | 100.0 | 77.8 |
| 208 | 96.3 | 69.4 | 76.2 | 88.9 | 22.2 | 94.4 | 74.4 |
| 209 | 100.0 | 47.2 | 81.0 | 77.8 | 48.1 | 100.0 | 74.4 |
| 210 | 100.0 | 58.3 | 11.1 | 66.7 | 14.8 | 44.4 | 44.0 |
| 211 | 92.6 | 72.2 | 23.8 | 69.4 | 33.3 | 44.4 | 52.2 |
| 212 | 100.0 | 47.2 | 85.7 | 83.3 | 33.3 | 100.0 | 74.9 |
| 213 | 100.0 | 50.0 | 71.4 | 83.3 | 44.4 | 77.8 | 70.5 |
| 214 | 96.3 | 58.3 | 60.3 | 69.4 | 51.9 | 38.9 | 63.3 |
| 215 | 96.3 | 50.0 | 47.6 | 75.0 | 29.6 | 38.9 | 56.0 |
| 216 | 96.3 | 52.8 | 66.7 | 88.9 | 48.1 | 27.8 | 66.2 |
| 217 | 92.6 | 66.7 | 17.5 | 86.1 | 3.7 | 11.1 | 45.4 |
| 218 | 100.0 | 58.3 | 90.5 | 94.4 | 70.4 | 94.4 | 84.5 |
| 219 | 92.6 | 66.7 | 14.3 | 88.9 | 22.2 | 27.8 | 48.8 |
| 220 | 96.3 | 52.8 | 68.3 | 80.6 | 29.6 | 83.3 | 67.6 |
| 221 | 92.6 | 77.8 | 52.4 | 86.1 | 44.4 | 27.8 | 64.7 |
| 222 | 96.3 | 61.1 | 38.1 | 97.2 | 48.1 | 27.8 | 60.4 |
| 223 | 81.5 | 41.7 | 7.9 | 38.9 | 66.7 | 44.4 | 39.6 |
| 224 | 100.0 | 80.6 | 81.0 | 88.9 | 59.3 | 38.9 | 78.3 |
| 225 | 96.3 | 47.2 | 36.5 | 86.1 | 25.9 | 27.8 | 52.7 |
| 226 | 100.0 | 75.0 | 90.5 | 91.7 | 29.6 | 88.9 | 81.2 |
| 227 | 92.6 | 83.3 | 58.7 | 94.4 | 77.8 | 27.8 | 73.4 |
| 228 | 100.0 | 44.4 | 79.4 | 86.1 | 22.2 | 94.4 | 71.0 |
| 229 | 96.3 | 47.2 | 55.6 | 88.9 | 40.7 | 22.2 | 60.4 |
| 230 | 96.3 | 50.0 | 55.6 | 77.8 | 22.2 | 27.8 | 57.0 |
| 231 | 100.0 | 55.6 | 98.4 | 77.8 | 33.3 | 100.0 | 79.2 |
| 232 | 100.0 | 63.9 | 98.4 | 77.8 | 44.4 | 100.0 | 82.1 |
| 233 | 96.3 | 69.4 | 90.5 | 97.2 | 22.2 | 44.4 | 75.8 |
| 234 | 96.3 | 77.8 | 90.5 | 94.4 | 18.5 | 44.4 | 76.3 |
| 235 | 100.0 | 66.7 | 14.3 | 80.6 | 22.2 | 27.8 | 48.3 |
| 236 | 100.0 | 66.7 | 77.8 | 86.1 | 11.1 | 27.8 | 67.1 |
| 237 | 100.0 | 38.9 | 77.8 | 80.6 | 3.7 | 27.8 | 60.4 |
| 238 | 100.0 | 61.1 | 79.4 | 77.8 | 18.5 | 38.9 | 67.1 |
| 239 | 100.0 | 69.4 | 77.8 | 80.6 | 11.1 | 27.8 | 66.7 |
| 240 | 100.0 | 66.7 | 76.2 | 86.1 | 51.9 | 66.7 | 75.4 |
| 241 | 44.4 | 22.2 | 15.9 | 47.2 | 11.1 | 38.9 | 27.5 |
| 242 | 100.0 | 50.0 | 19.0 | 69.4 | 22.2 | 38.9 | 45.9 |
| 243 | 96.3 | 61.1 | 6.3 | 80.6 | 14.8 | 27.8 | 43.5 |
| 244 | 100.0 | 58.3 | 88.9 | 94.4 | 48.1 | 55.6 | 77.8 |
| 245 | 92.6 | 52.8 | 23.8 | 83.3 | 40.7 | 22.2 | 50.2 |
| 246 | 100.0 | 61.1 | 33.3 | 58.3 | 77.8 | 22.2 | 56.0 |
| 247 | 96.3 | 44.4 | 20.6 | 91.7 | 48.1 | 77.8 | 55.6 |
| 248 | 96.3 | 44.4 | 25.4 | 86.1 | 48.1 | 27.8 | 51.7 |
| 249 | 96.3 | 36.1 | 55.6 | 63.9 | 59.3 | 22.2 | 56.5 |
| 250 | 100.0 | 72.2 | 57.1 | 94.4 | 59.3 | 22.2 | 69.1 |
| 251 | 96.3 | 47.2 | 57.1 | 75.0 | 63.0 | 100.0 | 68.1 |
| 252 | 92.6 | 63.9 | 66.7 | 88.9 | 66.7 | 22.2 | 69.6 |
| 253 | 100.0 | 33.3 | 50.8 | 69.4 | 33.3 | 22.2 | 52.7 |
| 254 | 77.8 | 69.4 | 20.6 | 66.7 | 59.3 | 22.2 | 49.8 |
| 255 | 100.0 | 69.4 | 17.5 | 66.7 | 70.4 | 22.2 | 53.1 |
| 256 | 96.3 | 66.7 | 23.8 | 75.0 | 40.7 | 50.0 | 54.1 |
| 257 | 100.0 | 44.4 | 92.1 | 80.6 | 70.4 | 94.4 | 80.2 |
| 258 | 96.3 | 36.1 | 54.0 | 83.3 | 51.9 | 22.2 | 58.5 |
| 259 | 88.9 | 41.7 | 87.3 | 77.8 | 48.1 | 94.4 | 73.4 |
| 260 | 100.0 | 58.3 | 82.5 | 88.9 | 63.0 | 72.2 | 78.3 |
| 261 | 96.3 | 69.4 | 27.0 | 83.3 | 63.0 | 22.2 | 57.5 |
| 262 | 96.3 | 63.9 | 58.7 | 88.9 | 44.4 | 83.3 | 70.0 |
| 263 | 100.0 | 97.2 | 28.6 | 91.7 | 48.1 | 22.2 | 62.8 |
| 264 | 100.0 | 61.1 | 90.5 | 94.4 | 59.3 | 88.9 | 83.1 |
| 265 | 88.9 | 80.6 | 81.0 | 83.3 | 70.4 | 88.9 | 81.6 |
| 266 | 96.3 | 55.6 | 79.4 | 91.7 | 48.1 | 22.2 | 70.5 |
| 267 | 92.6 | 47.2 | 84.1 | 88.9 | 59.3 | 22.2 | 71.0 |
| 268 | 92.6 | 66.7 | 81.0 | 86.1 | 77.8 | 27.8 | 75.8 |
| 269 | 100.0 | 63.9 | 88.9 | 88.9 | 44.4 | 5.6 | 72.9 |
| 270 | 100.0 | 75.0 | 11.1 | 86.1 | 11.1 | 22.2 | 47.8 |
| 271 | 100.0 | 75.0 | 79.4 | 86.1 | 44.4 | 22.2 | 72.9 |
| 272 | 92.6 | 66.7 | 15.9 | 72.2 | 37.0 | 66.7 | 51.7 |
| 273 | 92.6 | 50.0 | 28.6 | 75.0 | 63.0 | 27.8 | 53.1 |
| 274 | 100.0 | 47.2 | 87.3 | 88.9 | 66.7 | 88.9 | 79.7 |
| 275 | 96.3 | 80.6 | 96.8 | 86.1 | 81.5 | 94.4 | 89.9 |
| 276 | 100.0 | 77.8 | 81.0 | 88.9 | 70.4 | 100.0 | 84.5 |
| 277 | 100.0 | 72.2 | 54.0 | 72.2 | 29.6 | 22.2 | 60.4 |
| 278 | 92.6 | 27.8 | 19.0 | 50.0 | 37.0 | 22.2 | 38.2 |
| 279 | 100.0 | 66.7 | 77.8 | 86.1 | 70.4 | 94.4 | 80.7 |
| 280 | 96.3 | 66.7 | 55.6 | 86.1 | 48.1 | 22.2 | 64.3 |
| 281 | 81.5 | 55.6 | 61.9 | 75.0 | 66.7 | 22.2 | 62.8 |
| 282 | 92.6 | 80.6 | 85.7 | 94.4 | 63.0 | 61.1 | 82.1 |
| 283 | 81.5 | 58.3 | 12.7 | 63.9 | 33.3 | 0.0 | 40.1 |
| 284 | 96.3 | 44.4 | 60.3 | 77.8 | 59.3 | 22.2 | 61.8 |
| 285 | 77.8 | 58.3 | 11.1 | 72.2 | 51.9 | 22.2 | 44.9 |
| 286 | 70.4 | 30.6 | 27.0 | 66.7 | 59.3 | 22.2 | 44.0 |
| 287 | 85.2 | 80.6 | 49.2 | 75.0 | 59.3 | 66.7 | 66.7 |
| 288 | 85.2 | 72.2 | 46.0 | 77.8 | 85.2 | 50.0 | 66.7 |
| 289 | 100.0 | 80.6 | 92.1 | 91.7 | 85.2 | 83.3 | 89.4 |
| 290 | 92.6 | 63.9 | 47.6 | 75.0 | 81.5 | 55.6 | 66.2 |
| 291 | 88.9 | 75.0 | 74.6 | 75.0 | 66.7 | 38.9 | 72.5 |
| 292 | 88.9 | 47.2 | 82.5 | 80.6 | 66.7 | 66.7 | 73.4 |
| 293 | 85.2 | 66.7 | 74.6 | 77.8 | 63.0 | 66.7 | 72.9 |
| 294 | 100.0 | 75.0 | 68.3 | 77.8 | 81.5 | 83.3 | 78.3 |
| 295 | 88.9 | 50.0 | 39.7 | 77.8 | 51.9 | 77.8 | 59.4 |
| 296 | 88.9 | 61.1 | 33.3 | 75.0 | 59.3 | 61.1 | 58.5 |
| 297 | 70.4 | 63.9 | 27.0 | 58.3 | 51.9 | 22.2 | 47.3 |
| 298 | 81.5 | 52.8 | 41.3 | 83.3 | 66.7 | 38.9 | 58.9 |
| 299 | 92.6 | 36.1 | 66.7 | 75.0 | 55.6 | 38.9 | 62.3 |
| 300 | 85.2 | 88.9 | 12.7 | 77.8 | 22.2 | 22.2 | 48.8 |
| 301 | 96.3 | 61.1 | 79.4 | 80.6 | 37.0 | 83.3 | 73.4 |
| 302 | 100.0 | 94.4 | 93.7 | 86.1 | 77.8 | 83.3 | 90.3 |
| 303 | 63.0 | 52.8 | 44.4 | 47.2 | 59.3 | 22.2 | 48.8 |
| 304 | 100.0 | 77.8 | 100.0 | 94.4 | 59.3 | 83.3 | 88.4 |
| 305 | 100.0 | 52.8 | 87.3 | 72.2 | 51.9 | 72.2 | 74.4 |
| 306 | 81.5 | 33.3 | 63.5 | 80.6 | 55.6 | 66.7 | 62.8 |
| 307 | 96.3 | 75.0 | 84.1 | 91.7 | 66.7 | 77.8 | 82.6 |
| 308 | 92.6 | 75.0 | 88.9 | 86.1 | 70.4 | 72.2 | 82.6 |
| 309 | 92.6 | 77.8 | 88.9 | 83.3 | 63.0 | 72.2 | 81.6 |
| 310 | 100.0 | 88.9 | 95.2 | 86.1 | 70.4 | 94.4 | 89.9 |
| 311 | 85.2 | 44.4 | 61.9 | 86.1 | 66.7 | 27.8 | 63.8 |
| 312 | 96.3 | 36.1 | 54.0 | 86.1 | 51.9 | 50.0 | 61.4 |
| 313 | 96.3 | 75.0 | 71.4 | 88.9 | 63.0 | 77.8 | 77.8 |
| 314 | 100.0 | 69.4 | 84.1 | 86.1 | 66.7 | 83.3 | 81.6 |
| 315 | 92.6 | 66.7 | 90.5 | 88.9 | 59.3 | 66.7 | 80.2 |
| 316 | 63.0 | 52.8 | 36.5 | 72.2 | 59.3 | 22.2 | 50.7 |
| 317 | 66.7 | 58.3 | 54.0 | 66.7 | 51.9 | 22.2 | 55.6 |
| 318 | 66.7 | 33.3 | 17.5 | 66.7 | 48.1 | 22.2 | 39.6 |
| 319 | 81.5 | 75.0 | 63.5 | 86.1 | 70.4 | 27.8 | 69.6 |
| 320 | 100.0 | 77.8 | 92.1 | 83.3 | 66.7 | 83.3 | 85.0 |
| 321 | 92.6 | 69.4 | 57.1 | 72.2 | 70.4 | 22.2 | 65.2 |
| 322 | 96.3 | 61.1 | 65.1 | 69.4 | 48.1 | 55.6 | 66.2 |
| 323 | 100.0 | 97.2 | 96.8 | 91.7 | 63.0 | 77.8 | 90.3 |
| 324 | 100.0 | 75.0 | 95.2 | 97.2 | 63.0 | 94.4 | 88.4 |
| 325 | 81.5 | 63.9 | 9.5 | 66.7 | 11.1 | 16.7 | 39.1 |
| 326 | 66.7 | 55.6 | 34.9 | 69.4 | 48.1 | 55.6 | 52.2 |
| 327 | 92.6 | 50.0 | 82.5 | 80.6 | 63.0 | 38.9 | 71.5 |
| 328 | 100.0 | 41.7 | 85.7 | 80.6 | 66.7 | 22.2 | 71.0 |
| 329 | 70.4 | 33.3 | 15.9 | 47.2 | 33.3 | 22.2 | 34.3 |
| 330 | 81.5 | 19.4 | 22.2 | 63.9 | 11.1 | 44.4 | 37.2 |
| 331 | 96.3 | 44.4 | 23.8 | 80.6 | 63.0 | 72.2 | 56.0 |
| 332 | 100.0 | 55.6 | 49.2 | 58.3 | 74.1 | 44.4 | 61.4 |
| 333 | 100.0 | 91.7 | 88.9 | 91.7 | 70.4 | 100.0 | 89.9 |
| 334 | 88.9 | 55.6 | 47.6 | 83.3 | 74.1 | 22.2 | 61.8 |
| 335 | 92.6 | 27.8 | 30.2 | 69.4 | 48.1 | 38.9 | 47.8 |
| 336 | 88.9 | 55.6 | 85.7 | 86.1 | 70.4 | 66.7 | 77.3 |
| 337 | 92.6 | 44.4 | 82.5 | 83.3 | 63.0 | 66.7 | 73.4 |
| 338 | 85.2 | 41.7 | 60.3 | 72.2 | 51.9 | 66.7 | 61.8 |
| 339 | 88.9 | 47.2 | 81.0 | 80.6 | 63.0 | 66.7 | 72.5 |
| 340 | 100.0 | 91.7 | 82.5 | 91.7 | 81.5 | 83.3 | 87.9 |
| 341 | 100.0 | 77.8 | 23.8 | 77.8 | 48.1 | 22.2 | 55.6 |
| 342 | 100.0 | 72.2 | 93.7 | 88.9 | 74.1 | 83.3 | 86.5 |
| 343 | 77.8 | 58.3 | 74.6 | 69.4 | 59.3 | 94.4 | 71.0 |
| 344 | 92.6 | 44.4 | 52.4 | 66.7 | 63.0 | 88.9 | 63.3 |
| 345 | 88.9 | 61.1 | 50.8 | 63.9 | 63.0 | 83.3 | 64.3 |
| 346 | 88.9 | 50.0 | 42.9 | 77.8 | 63.0 | 38.9 | 58.5 |
| 347 | 81.5 | 55.6 | 38.1 | 75.0 | 51.9 | 61.1 | 57.0 |
| 348 | 66.7 | 44.4 | 42.9 | 69.4 | 51.9 | 50.0 | 52.7 |
| 349 | 96.3 | 52.8 | 39.7 | 63.9 | 55.6 | 83.3 | 59.4 |
| 350 | 88.9 | 44.4 | 57.1 | 52.8 | 55.6 | 50.0 | 57.5 |
| 351 | 81.5 | 38.9 | 38.1 | 63.9 | 55.6 | 22.2 | 49.3 |
| 352 | 70.4 | 36.1 | 11.1 | 58.3 | 44.4 | 5.6 | 35.3 |
| 353 | 81.5 | 41.7 | 42.9 | 63.9 | 48.1 | 50.0 | 52.7 |
| 354 | 100.0 | 77.8 | 85.7 | 91.7 | 55.6 | 94.4 | 84.1 |
| 355 | 96.3 | 61.1 | 69.8 | 83.3 | 55.6 | 72.2 | 72.5 |
| 356 | 100.0 | 91.7 | 30.2 | 88.9 | 59.3 | 22.2 | 63.3 |
| 357 | 85.2 | 69.4 | 68.3 | 66.7 | 55.6 | 88.9 | 70.5 |
| 358 | 74.1 | 69.4 | 73.0 | 77.8 | 70.4 | 83.3 | 73.9 |
| 359 | 81.5 | 66.7 | 39.7 | 69.4 | 48.1 | 27.8 | 55.1 |
| 360 | 92.6 | 72.2 | 87.3 | 77.8 | 66.7 | 88.9 | 81.2 |
| 361 | 85.2 | 86.1 | 11.1 | 77.8 | 55.6 | 22.2 | 52.2 |
| 362 | 81.5 | 47.2 | 76.2 | 72.2 | 55.6 | 72.2 | 68.1 |
| 363 | 100.0 | 77.8 | 85.7 | 77.8 | 51.9 | 94.4 | 81.2 |
| 364 | 81.5 | 41.7 | 36.5 | 61.1 | 44.4 | 72.2 | 51.7 |
| 365 | 96.3 | 61.1 | 77.8 | 77.8 | 51.9 | 83.3 | 74.4 |
| 366 | 100.0 | 61.1 | 71.4 | 72.2 | 40.7 | 22.2 | 65.2 |
| 367 | 96.3 | 69.4 | 60.3 | 66.7 | 51.9 | 50.0 | 65.7 |
| 368 | 96.3 | 58.3 | 73.0 | 77.8 | 51.9 | 77.8 | 72.0 |
| 369 | 92.6 | 55.6 | 69.8 | 61.1 | 51.9 | 88.9 | 68.1 |
| 370 | 96.3 | 47.2 | 69.8 | 58.3 | 51.9 | 77.8 | 65.7 |
| 371 | 92.6 | 72.2 | 60.3 | 63.9 | 63.0 | 33.3 | 65.2 |
| 372 | 92.6 | 75.0 | 58.7 | 75.0 | 66.7 | 50.0 | 69.1 |
| 373 | 96.3 | 50.0 | 68.3 | 66.7 | 55.6 | 44.4 | 64.7 |
